# The histone H4 proteoform dynamics in response to SUV4-20 inhibition reveals single molecule mechanisms of inhibitor resistance

**DOI:** 10.1186/s13072-018-0198-9

**Published:** 2018-06-07

**Authors:** Tao Wang, Matthew V. Holt, Nicolas L. Young

**Affiliations:** 1 Verna and Marrs McLean Department of Biochemistry and Molecular Biology, Baylor College of Medicine, Houston, TX USA; 2 Department of Molecular and Cellular Biology, Baylor College of Medicine, Houston, TX USA

**Keywords:** Histone methyltransferase, SUV4-20, Histone post-translational modifications, Epigenetic inhibitor, Breast cancer, Top-down proteomics

## Abstract

**Background:**

The dynamics of histone post-translational modifications (PTMs) are sparsely described, especially in their true physiological context of proteoforms (single histone molecules harboring combinations of PTMs).

**Methods:**

Here we time-resolve the response of cells to SUV4-20 methyltransferase inhibition and unbiasedly quantitate the dynamic response of histone H4 PTMs and proteoforms.

**Results:**

Contrary to the prevailing dogma, cells exhibit an immediate-early response with changes to histone proteoforms. Cells also recover to basal-like conditions upon removal of epigenetic inhibitors rapidly. Inhibition of SUV4-20 results in decreased H4{K20me2}; however, no effects on H4{K20me3} are observed, implying that another enzyme mediates H4K20me3. Most surprisingly, SUV4-20 inhibition results in an increase in histone H4 acetylation attributable to proteoforms containing K20me2. This led us to hypothesize that hyperacetylated proteoforms protect K20me2 from demethylation as an evolved compensatory mechanism. This concept is supported by subsequent results that pretreatment with an HDACi substantially diminishes the effects of SUV4-20 inhibition in prone cells and is further confirmed by HATi-facilitating SUV4-20 inhibition to decrease discrete H4{K20me2} in resistant cells.

**Conclusions:**

The chromatin response of cells to sudden perturbations is significantly faster, nuanced and complex than previously described. The persistent nature of chromatin regulation may be achieved by a network of dynamic equilibria with compensatory mechanisms that operate at the proteoform level.

**Electronic supplementary material:**

The online version of this article (10.1186/s13072-018-0198-9) contains supplementary material, which is available to authorized users.

## Background

Histone PTMs were first described in the middle of the last century [1]. The ‘histone code hypothesis’ is the concept where histone modifications serve as binding sites for regulatory proteins and result in specific biological functions [2, 3]. The recruitment of regulatory proteins is widely accepted as the primary role of histone PTMs. The current evidence suggests that there is a moderately strong, but not strictly injective (one-to-one) relationship between discrete histone PTMs and specific biological function [4, 5].

Histone modifications do not function in isolation. Increasing evidence suggests that histone PTMs routinely function in combination with each other, and these combinations are more specific to individual biological processes than discrete PTMs [6, 7]. For example, 53BP1 prefers to bind to nucleosomes with H4K20me2 but is hindered by the presence of H4K16ac on the same molecule [8]. Considering that H4K20me2 is nominally 70–90% abundant per histone H4 molecule and there are two histone H4 molecules per nucleosome, there is on average more than one H4K20me2 per nucleosome [9, 10] (Also see Table 1). This makes any independent function of this mark, without consideration of co-occurring PTMs, difficult to explain. This indicates that histone PTMs do not only exert their function individually but also in a combinatorial manner. Thus, to better understand the role of histone PTMs, it is necessary to investigate the combinations of PTMs on single histone molecules or ‘proteoforms’ for biological function.

**Table 1:** Relative basal abundance of discrete H4 PTMs

	SUM159 cells (%)	MCF7 cells (%)
Nα-ac	98.2 ± 0.6	96.4 ± 0.9
S1ph	0.1 ± 0.0	0.2 ± 0.1
R3me1	0.2 ± 0.1	0.2 ± 0.1
K5ac	2.0 ± 0.4	3.2 ± 0.7
K8ac	1.7 ± 0.6	1.1 ± 0.2
K12ac	6.0 ± 1.4	3.3 ± 0.6
K16ac	40.6 ± 2.4	35.8 ± 0.9
K20me1	12.2 ± 1.3	7.7 ± 0.6
K20me2	76.2 ± 1.6	78.7 ± 1.3
K20me3	8.3 ± 1.1	10.1 ± 0.7
K20un	3.3 ± 1.1	3.4 ± 0.8
K31ac	7.2 ± 1.1	7.5 ± 0.8

Histone PTM reaction kinetics are mediated by several factors that implicitly influence histone PTM dynamics. Firstly, the kinetics are modulated by the abundance, localization, and activity of the enzymes that ‘write’ and ‘erase’ modifications [11]. Secondly, the kinetics are controlled by the availability of substrates. Increasing SAM (S-adenosyl methionine), the substrate of methyltransferases that provides the methyl group increases histone methylation, for example resulting in an increase of H3K4me3 in 2 h [12]. Finally, the modification site itself helps determine the kinetics of modification [13].

Relatively high turnover of histone PTMs are observed even under steady-state growth conditions and steady abundance of PTMs [11]. This results from equal ‘writing’ and ‘erasing’ of PTMs over time. Turnover rates are modulated by many biological processes. For example, fast turnover rates indicate that a PTM is likely associated with active gene transcription regions, such as H3K4me3 and H3K36me3. However, even marks associated with repression of transcription exhibit continuous turnover. The ongoing turnover of histone PTMs implies a potentially important role of the dynamics (time-resolved change, not integrated over time) of histone PTMs in fundamental chromatin biology. However, dynamics of histone PTMs has not been well explored. Here we go several steps further to reveal how the inhibition of the enzyme(s) that mediate one given discrete histone PTM dynamically affects all other PTMs on the single molecule level.

Antibody-based assays, such as western blots and ELISA assays, have been used to measure the dynamics of histone modifications, but suffer from multiple critical deficiencies [12, 14, 15]. Antibodies are primarily generated to site specifically recognize a single histone PTM, such as acetylation, methylation and phosphorylation. However, the specificity of these antibodies is dependent on any neighboring PTMs that may obfuscate recognition via epitope occlusion [16]. It is challenging to distinguish different methylation states (me1, me2, and me3) on the same residue. Even if a tenfold specificity toward one methylation state over another is achieved in vitro, an in vivo difference in abundance approaching tenfold, as is the case for the H4K20me2/me3 comparison, obliterates the capacity to independently study these PTMs. Thus, it is critical to robustly detect combinations of histone PTMs even if only for the accurate quantitative study of discrete PTMs free from biases emanating from neighboring PTMs. Similarly, it is imperative to discriminate precise methylation states on the same position as these likely carry distinct functions.

Bottom-up MS/MS methods have been developed to precisely distinguish and quantitate histone PTMs, but are limited [17–20]. Bottom-up MS/MS analyses make use of proteases to generate peptides that are amenable to chromatographic separation and gas phase sequencing. These methods are quantitative, high throughput, relatively unbiased and free from epitope occlusion. The primary limitation of bottom-up MS/MS is the inability to analyze distally co-occurring PTMs on the single molecule level due to the use of proteases. Middle-down proteomics uses more selective proteolysis to yield enhanced single molecule PTMs connections but only between nearby PTMs [21]. As explained above, the quantitative study of distally co-occurring PTMs is essential for a complete understanding of the functions of histone PTMs.

We have developed a novel top-down LC–MS/MS proteomics method to quantitatively study the full proteoforms of histone H4. Top-down proteomics forgoes proteolysis. Instead, this method analyzes the intact protein and proteoforms, thus preserving the molecular connectivity between distal sites of variable modification. Top-down proteomics presents many technical challenges that have largely limited such approaches. Here we use our recently developed method that overcomes these limitations to yield high throughput, robust, reproducible and highly quantitative analysis of histone H4 proteoforms. This method chromatographically separates intact histone proteoforms and uses electron transfer dissociation-based top-down fragmentation to quantitate histone proteoforms. From this data, we can retrieve PTM combinations at multiple levels (e.g. discrete PTMs, binary PTM combinations, ternary PTM combinations, higher order combinations, up to and including full proteoforms) and reveal a network between histone PTMs on single molecules.

Here, we study the dynamics of H4K20me2 in response to the sudden inhibition of the specific upstream methyltransferase ‘writer’ and characterize the cross-talk between K20me2 and other histone PTMs on histone H4. H4K20 is di- and possibly tri-methylated by two homologous histone methyltransferases (SUV4-20H1 and SUV4-20H2) [22]. It has been proposed that K20 is monomethylated by SET8 and subsequently modified by SUV4-20 [23–26]. Double knockout of SUV4-20H1/H2 is lethal in mice, which results in loss of both H4K20me2 and K20me3 [9]. This supports the idea that SUV4-20H1 and SUV4-20H2 are methyltransferases for K20me2 and possibly K20me3 within the limits of the technology. However, biochemical data suggest that neither SUV4-20H1 nor SUV4-20H2 deposits K20me3 efficiently in vitro [27]. Decreases in K20me3 may simply be due to decreases in K20me2, particularly if K20me3 is exclusively generated from K20me2. This concept is analogous to the evidence that SUV4-20H2 only generates K20me2 from K20me1 [28]. Thus, it remains unknown if SUV4-20H1/H2 is required for H4K20me3. Our results indicate that SUV4-20H1/H2 likely does not directly mediate K20me3 in living cells or does so much less dynamically than K20me2.

We present data of an unconventional level of detail at multiple overlapping and interrelated levels. This necessitates the use of novel nomenclature and notation to adequately describe and sufficiently distinguish different interrelated concepts of molecular identity concisely. For example, ‘the discrete PTM H4K20me2’ is indicated concisely by simply: H4{K20me2}. (This implies nothing about other sites of variable modification.) This is necessary to distinguish it from the proteoform that contains dimethylation at lysine 20, indicated by H4<K20me2>. (This declares that all other sites of variable modification are definitively unmodified). In the same way, discrete binary and ternary combinations are quantitated, which is simply denoted as H4{PTM1, PTM2} and H4{PTM1, PTM2, PTM3}, respectively. It may be helpful to understand that ‘<>’ represents a sparse vector while ‘{}’ represents a set (Additional file 1). At times, we also refer to generic PTMs without designation of location.

In this study, full H4 proteoforms were quantitated in two breast cancer cell lines (SUM159 and MCF7). The dynamics of the cross-talk between H4K20me2 and other PTMs were quantitatively characterized in response to the application of a recently developed SUV4-20 inhibitor (A-196). A-196 (6,7-dichloro-*N*-cyclopentyl-4-(pyridin-4-yl)) is a selective chemical probe for SUV4-20, which competes with substrate binding [29]. We find that K20me2 is regulated in an unprecedentedly dynamic and remarkably nuanced way, while SUV4-20 is not associated with K20me3. In addition, hyperacetylated proteoforms with K20me2 are resistant to demethylation. Overall, this work provides novel insights into H4 proteoforms and the surprisingly dynamic interplay between PTMs on single H4 molecules in response to the inhibition of specific epigenetic enzymes.

## Results

### Cell lines differ only slightly in basal histone H4 epigenetic state

**Fig. 1:**
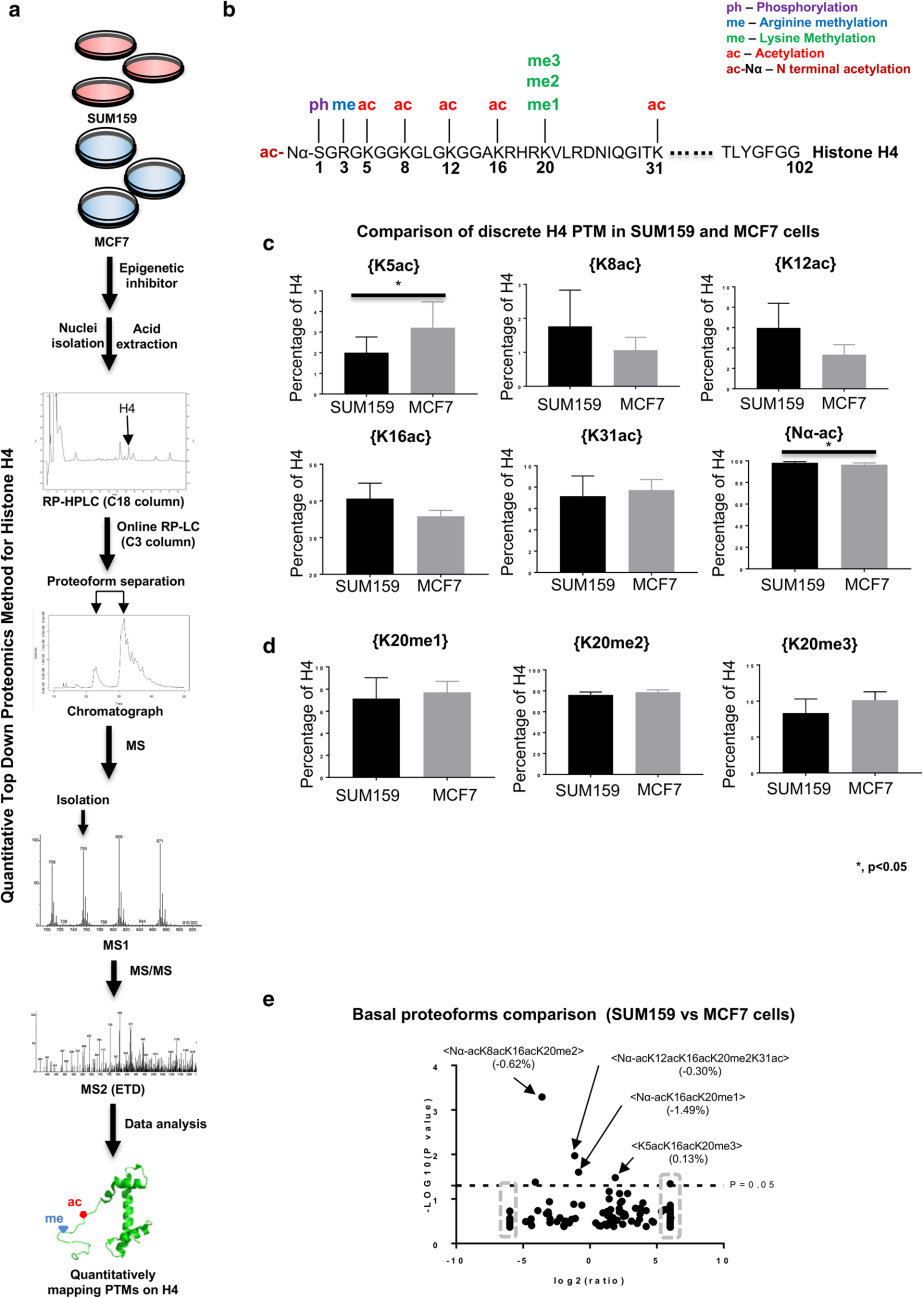
SUM159 and MCF7 cells differ in basal histone H4 epigenetic states. **a** Flowchart of experimental setup, **b** selected histone PTMs of the *N*-terminal tail of H4, **c** comparison of discrete H4 acetylations between SUM159 and MCF7 cells. *p < 0.05. **d** Discrete level of K20 methylation states are similar between the selected cell lines. **e** Volcano plot of proteoforms differences between these two cell lines. Data points in the gray dashed squares indicate infinity fold change. Error bars in **c** and **d** represent standard error from three biological replicates

Later in the manuscript, the stark differences in the dynamic responses of SUM159 and MCF7 cell lines to SUV4-20 inhibition become apparent. Thus, we establish here that SUM159 and MCF7 cells have similar basal abundances of discrete PTMs and binary combinations and proteoforms, with some notable but minor differences. A relatively extensive set of histone H4 PTMs are characterized in this study (Fig. 1a, b). Two discrete acetylations are statistically significantly different: N-terminal acetylation H4{Nα-ac} and H4{K5ac} (Student’s *t* test *p* < 0.05). H4{Nα-ac} is slightly higher and H4{K5ac} is lower in SUM159 cells compared to MCF7 cells (Fig. 1c; Table 1). No significant discrepancy in discrete H4K20 methylation status is observed between these two cell lines (Fig. 1d). SUM159 and MCF7 cells differ in the abundance of some proteoforms (the unique combinations of PTMs on single H4 molecules) (Fig. 1e; Additional file 1: Table S1). Over 200 proteoforms are identified in these two cells. In summary, these two cell lines diverge for a few proteoforms and the discrete histone PTMs are very similar. Thus, slight differences in proteoforms or combinations of PTMs and not discrete histone PTMs distinguish these cell lines. The real differences between these cell lines are revealed later to be in the dynamics of these PTMs and proteoforms. This is not reflected in steady-state measurements.

### H4K20me2 is immediately affected by SUV4-20 inhibition at both the discrete and proteoform levels in SUM159 cells

**Fig. 2:**
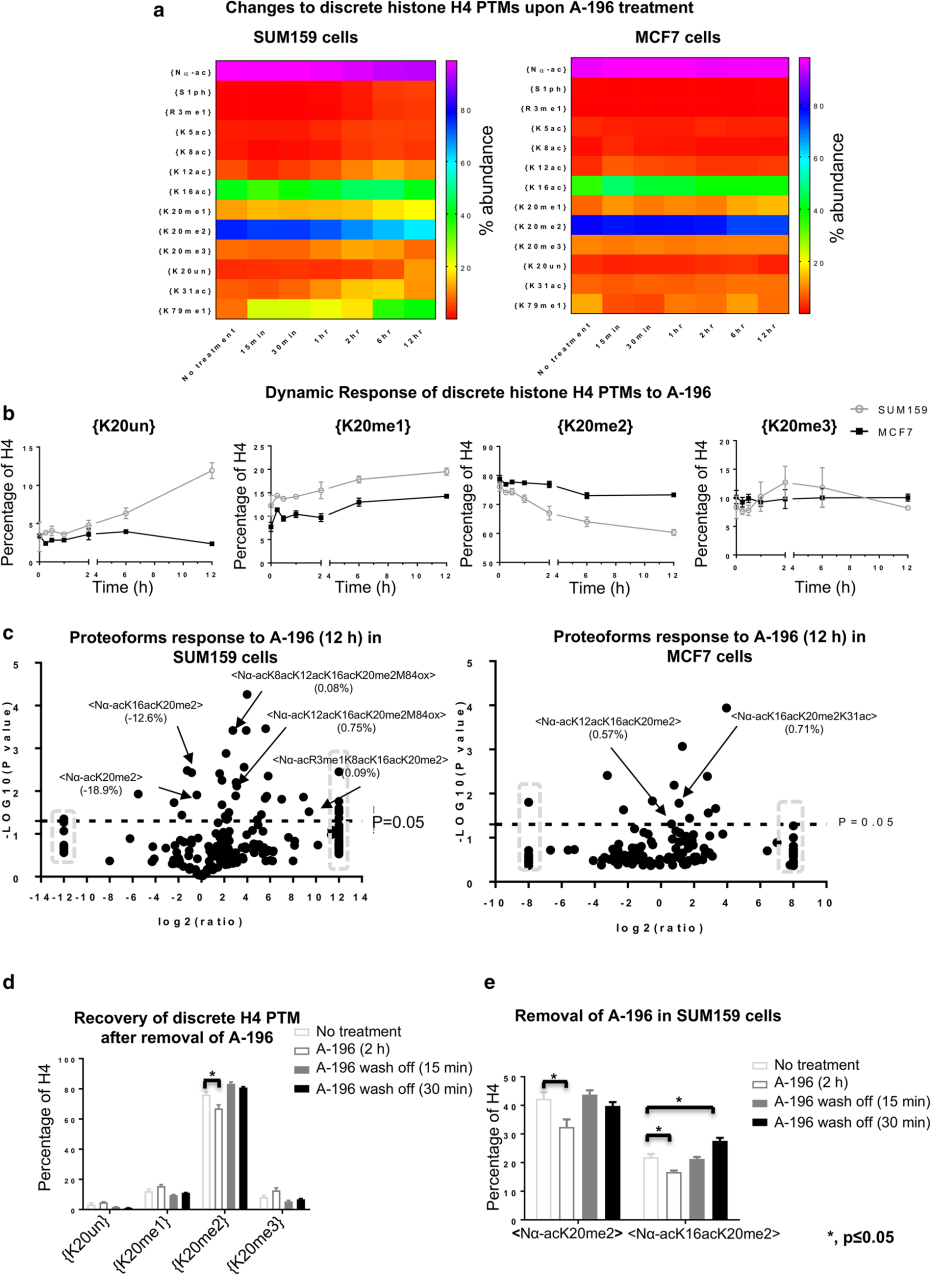
Cells respond to SUV4-20 inhibition immediately and recover rapidly after removal of SUV4-20 inhibitor. **a** Heatmaps of the effect of SUV4-20 inhibition on the relative abundance of all discrete PTMs in SUM159 and MCF7 cells. **b** Relative abundance of discrete K20 methylation responds to SUV4-20 inhibition in SUM159 and MCF7 cells. **c** Volcano plot of changes in the relative abundance of proteoforms due to 12-h SUV4-20 inhibition in SUM159 cells (left panel) and MCF7 cells (right panel). Data points in the gray dashed squares indicate infinity fold change. **d** The level of H4{K20me2} recovers in 15 min after removal of SUV4-20 inhibitor in SUM159 cells. **e** Two selected decreased proteoforms in SUM159 cells, due to 2 h SUV4-20 inhibition, also recover in 15 min after removal of SUV4-20 inhibitor. *p < 0.05. Error bars in **b**, **d**, **e** represent standard error from three biological replicates

SUM159 cells are prone to the effects of SUV4-20; however, later we show that MCF7 cells are resistant to this treatment and reveal the single molecule mechanisms that explain this difference. Thus, we show here the extent, timescale and proteoform level details of the changes induced in SUM159 cells for later comparison. In SUM159 cells, discrete H4{K20me2} is markedly affected immediately upon SUV4-20 inhibition and decreases during the time course. Discrete H4{K20me2} decreases in 15 min and continuously decreases post-SUV4-20 inhibition (Fig. 2a, b). After 12 h of A-196 treatment, discrete H4{K20me2} decreases from 76.2% prior to treatment to 60.4%. Less than twofold loss in abundance may be arbitrarily considered as a nonsignificant change in many studies; however, discrete H4{K20me2} is a very abundant PTM. A twofold decrease of this marker may be lethal and a twofold increase is impossible. Thus, only considering fold change of PTMs may be misleading. Discrete H4{K20me2} decreases very rapidly in the first 6 h of treatment, but the rate of decrease slows in the 6–12 h time frame. The loss of H4{K20me2} results in increased H4{K20me1}. This recapitulates that H4K20me1 is the substrate for SUV4-20 [27, 30].

In SUM159 cells, two proteoforms containing K20me2 are decreased significantly upon SUV4-20 inhibition. By 12 h, H4<Nα-acK20me2> and H4<Nα-acK16acK20me2> are reduced by 18.9% points (from 42.3 to 23.4%) and 12.5% points (from 21.8 to 9.3%) (in total 31.4% points combined), respectively (Fig. 2c, Additional file 1: S1A; Table S2), while the total loss of H4{K20me2} is 15.8% points (from 76.2 to 60.4%). Note that H4{K20me2} is an ensemble average of many proteoforms, and we show later that some of the loss of these proteoforms is due to acetylation not demethylation. H4<Nα-acK20me2> and <Nα-acK16acK20me2> begin decreasing as quickly as in 15 min upon SUV4-20 inhibition (Additional file 1: Fig. S1A). The limitations of previous technologies may prejudice some readers to assume that because some of these are small fold changes (e.g., from 76.2 to 60.4%) that they are not meaningful. These changes are statistically significant (Additional file 1: Table S2). Post-translational modification and proteoform abundances are far from uniformly distributed (Table 1, Additional file 1: Table S2). Thus, the scale of the change is absolute terms can be astoundingly immense with small fold change if the PTM or proteoform is abundant. A change to 10–30% of total chromatin is indeed very dramatic, requiring the expenditure of massive amounts of energy.

### H4K20me2 is promptly and dramatically affected by SUV4-20 inhibition at proteoform level, but not at the discrete PTM level in MCF7 cells

In the previous section, we established the dynamics and extent of changes induced by SUV4-20 in the more prone SUM159 cell line. Here we show that MCF7 cells behave very differently in both timescale, extent and which proteoforms are affected. Later, we establish how these differences explain why this cell line is resistant to SUV4-20 inhibition and how to manipulate the underlying mechanisms of resistance.

In MCF7 cells, discrete H4{K20me2} is only slightly decreased after SUV4-20 inhibition (Fig. 2a, b), which is decreased from 78.7 to 73.3% after 12 h post-A-196 application. This decrease is mainly observed in the 2–12 h time frame. This slight decrease of H4{K20me2} also results in the increase of H4{K20me1} as in SUM159 cells. The diverse response to SUV4-20 inhibition in these two cell lines indicates that H4{K20me2} is regulated through more complex mechanisms explored below. MCF7 cells exhibit an extremely rapid, but diffuse and transient response to SUV4-20 inhibition at the proteoform level. Although there is no apparent change to discrete H4{K20me2} for at least 2 h, at 15 min post-A-196 application the H4<Nα-acK20me2> proteoform transiently decreases by 5.2% points (from 44.6 to 39.4%) (Additional file 1: Fig. S1A, left panel; Table S3). This proteoform is transiently affected by SUV4-20 inhibition and gradually reverts toward the basal level. This inexorably causes one proteoform containing K20me1, H4<Nα-acK20me1>, to increase transiently. The H4<Nα-acK20me2> proteoform is only decreased by 1.8% points (from 44.6 to 42.8%) after 12-h treatment (Fig. 2c, right panel, Additional file 1: S1A, left panel; Table S4).

### Cells recover rapidly after removal of SUV4-20 inhibitor

Above we show though several examples that changes to histone PTMs and proteoforms can be extraordinarily rapid upon application of an epigenetic inhibitor. This peaked our curiosity as to how rapidly cells would respond to the removal of epigenetic inhibitor. Not only does this have wider implications in clinical pharmacology, later we use a combinatorial inhibitor approach, sometimes sequentially, to validate our model of the difference in response between cell lines. Thus, we establish here this off-rate dynamic.

Upon removal of the SUV4-20 inhibitor from media, cells recover in their histone H4 status to basal levels in as quickly as 15 min. Upon observing the remarkably quick response to SUV4-20 inhibition, we wondered if histone PTMs, or more specifically if H4K20 methylation, are inherently capable of rapid dynamic changes in cells. If so the recovery of K20me2 after removal of inhibitor might be similarly as rapid. Since A-196 keeps affecting SUM159 cells during the whole time course, SUM159 cells were treated with A-196 for 2 h. After removing A-196, discrete H4{K20me2} completely recovers within 15 min (Fig. 2d). The H4{K20me1} level is also restored to the basal level. This further recapitulates that K20me1 is the substrate for SUV4-20. Proteoforms affected by SUV4-20 inhibition also recover within 15 min (Fig. 2e). The <Nα-acK20me2> and <Nα-acK16acK20me2> proteoforms that are markedly decreased at 2 h after SUV4-20 inhibition and are persistently decreased after 12 h (Additional file 1: Fig. S1A and B, left panel; Table S5). Remarkably, they are restored to basal abundances within 15 min after removing SUV4-20 inhibitor (Fig. 2e and Additional file 1: S1C; Table S6). After 30 min, <Nα-acK16acK20me2> overshoots and is increased slightly (*p* < 0.05) (Fig. 2e, Additional file 1: S1D; Table S7). However, nearly all other proteoforms are restored to basal levels. These remarkably quick and dynamically fluctuating recoveries argue for a dynamic equilibrium model.

In summary, epigenetic marks that are remarkably stable over long periods of time may be maintained through active and dynamic regulation of histone PTMs and evolved compensatory mechanisms. Cells respond to at least this epigenetic inhibitor within minutes upon application and recover immediately to their basal level after removal of inhibitor. This indicates a dynamic equilibrium model of chromatin and histone modifications. Such a dynamic equilibrium model of chromatin epigenetic marks is further supported by other recent findings that also indicate evolved dynamic compensatory responses to epigenetic inhibitors [31].

### H4K20me3 is not decreased upon SUV4-20 inhibition

SUV4-20 has been previously attributed to mediate H4K20me3; however, the validity of this attribution has been challenged by other laboratories. Thus, the effects of SUV4-20 were initially central to our expected results. We show here that our data are supportive of the converse hypothesis that SUV4-20 does not mediate this modification. Thus, we establish here the irrelevance of K20me3 and later focus on H4K20me2.

The abundance of discrete H4{K20me3} is not decreased in either cell line at any time within 12 h of treatment, despite strong evidence that A-196 results in the decrease of K20me3 at least on longer timescales [29]. Conversely, discrete H4{K20me3} in SUM159 and MCF7 cells is not affected upon SUV4-20 inhibition (Fig. 2b).

### SUV4-20 inhibition results in anti-correlation in abundance between histone H4 acetylation and K20me2

Having established the early temporal dynamics, methylation degree modulated by SUV4-20 inhibition, and differences between cell lines, we next look beyond K20 methylation and ask how SUV4-20 inhibition affects other PTMs. We show here that to our surprise that acetylation is dramatically increased as K20me2 decreases. In the next section, we will show that despite this anti-correlation in abundance the increases in acetylation abundance are remarkably mostly on molecules containing K20me2 and later that this is central to mechanisms of resistance to SUV4-20 inhibition.

**Fig. 3:**
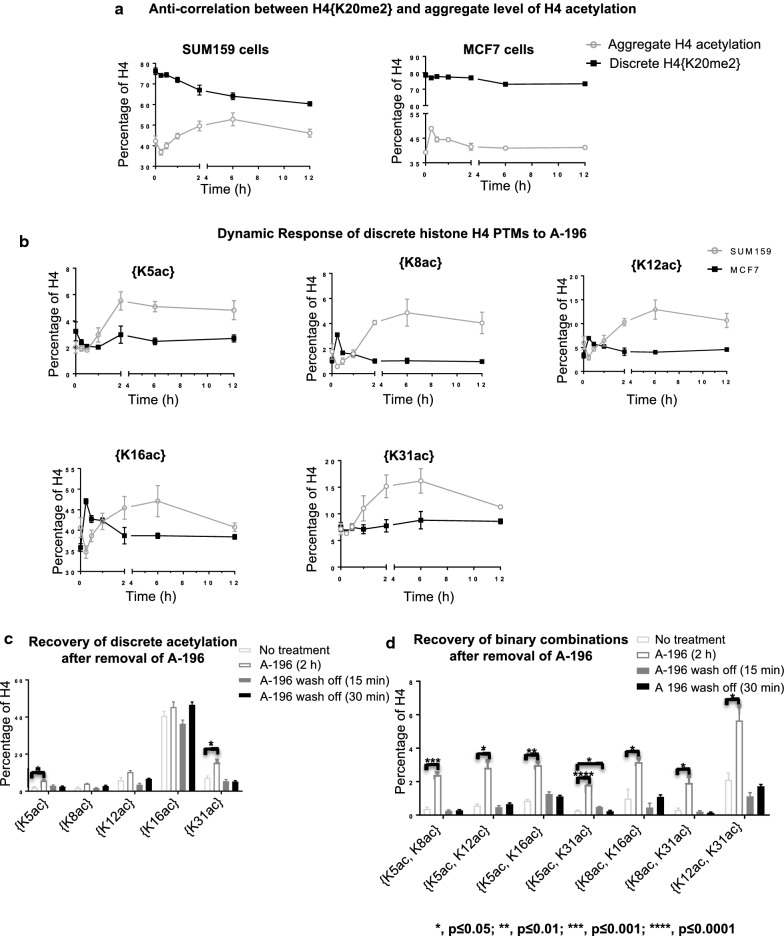
H4K20me2 and H4 acetylation are anti-correlated in abundance. **a** The effect of SUV4-20 inhibition on discrete H4{K20me2} and total level of H4 acetylation. **b** The effect of SUV4-20 inhibition on discrete H4 acetylations in SUM159 and MCF7 cells. **c** Increased discrete H4 acetylation and **d** binary combinations of H4 acetylation upon SUV4-20 inhibition recover within 15 min after removal of SUV4-20 inhibitor in SUM159 cells. *p < 0.05; **p < 0.01; ***p < 0.001, *p* < 0.0001. Error bars in **a**, **b**, **c**, **d** represent standard error from three biological replicates

The abundance of total acetylation is anti-correlated with H4{K20me2}, upon SUV4-20 inhibition. Total level of H4 acetylation is gradually increased as {K20me2} is decreased (Fig. 3a, left panel). As H4{K20me2} slowly decreases in the 6–12 h time frame, the rate of increase in H4 acetylation decelerates as well in SUM159 cells. In MCF7 cells, the total abundance of H4 acetylation is quickly, dramatically and transiently increased in 15 min upon A-196 application, although H4{K20me2} is not affected significantly at this time (Fig. 3a, right panel). This is the case only at the discrete level, not at the proteoform level.

In SUM159 cells, SUV4-20 inhibition significantly increases discrete histone H4 acetylation after 2 h, as discrete H4{K20me2} is dramatically decreased (Figs. 2b, 3b). Discrete H4{K5ac} and {K31ac} both significantly increase across the 2–12 h timescale post-SUV4-20 inhibition (*p* < 0.05). The abundance of discrete H4{K8ac}, {K12ac} and {K16ac} clearly trends toward increasing in a manner similar to the other H4 acetylations; however, they narrowly do not meet statistical criteria for change (*p* = 0.056 {K8ac}, *p* = 0.069 {K12ac}, *p* = 0.054 {K16ac} at 2 vs. 0 h). H4{K5ac}, {K8ac}, {K12ac} and {K16ac} reach their plateau after 6-h treatment. Single molecule binary combinations of H4 acetylation also increase in SUM159 cells after 12-h treatment (Additional file 1: Fig. S2A and B).

Intriguingly, SUV4-20 inhibition results in a quick, but transient, increase of histone H4 discrete acetylation within 15 min in MCF7 cells (Fig. 3b). Discrete H4{K8ac}, {K12ac} and {K16ac} increase in 15 min after SUV4-20 inhibition (*p* < 0.05 for K8ac and K12ac, *p* < 0.01 for K16ac). These acetylations then return to the basal level after 2 h A-196 application. Binary combinations of H4 acetylation significantly increase in MCF7 cells after 15-min treatment as well (Additional file 1: Fig. S2A and C).

SUM159 and MCF7 cells respond to SUV4-20 inhibition differently, notably in the changes in acetylation. Histone H4 acetylations increase at a slower rate in SUM159 cells, compared to MCF7 cells. The increase of histone acetylation in SUM159 cells is observed primarily after 2 h SUV4-20 inhibition, whereas the increase in histone acetylation in MCF7 cells is primarily an unsustained, transient increase in the 15–30 min time frame. This led us to later explore if this causes the different response dynamics of K20me2 to SUV4-20 inhibition in these two cell lines.

### Recovery from SUV4-20 inhibition results in the rapid decrease of H4 acetylation levels, further confirming the anti-correlation in abundance between K20me2 and acetylation

Having shown anti-correlation in the abundance of K20me2 and histone H4 acetylation upon SUV4-20 inhibition, we show here that this anti-correlation in abundance remains upon removal of the SUV4-20 inhibitor. This further confirms this phenomenon helps establish it as a fundamental feature of the cell. Later we show that this feature is central to the capacity of cells to resist changes to their epigenetic state.

The effect of SUV4-20 inhibition on all discrete and binary combinations of H4 acetylation disappears within 15 min in SUM159 cells. The discrete PTMs: H4{K5ac} and H4{K31ac} are both statistically significantly increased after 2 h of A-196 treatment. Discrete H4{K8ac} (*p* = 0.054 at 2 h vs. 0 h), {K12ac} (*p* = 0.069 at 2 h vs. 0 h) and {K16ac} are also increased (*p* = 0.054 at 2 h vs. 0 h), but narrowly do not pass the arbitrary statistical threshold of *p* < 0.05 (Fig. 3b). After removal of A-196, all discrete acetylations recover to the basal level immediately (Fig. 3c). The binary H4 acetylation states that are increased in 2 h upon SUV4-20 inhibition nearly all decrease to basal levels within 15 min after removal of A-196 (Additional file 1: Fig. S2D and 3D). The strong temporal correlation both upon application and removal of SUV4-20 inhibitor further suggests that H4 acetylation is mechanistically linked to K20me2.

### H4K20me2 is positively correlated with H4 acetylation on the single molecule level

Having established the anti-correlation in abundance between H4K20me2 and histone H4 acetylation, we show here that while these modifications are indeed divergent in their velocities the increases in acetylation are largely on H4K20me2-containing molecules. This is a counterintuitive relationship that surprised us as well. As we show later, this surprising relationship is essential to the mechanisms of resistance and that with sufficient understanding of the single molecule biochemistry it is possible to manipulate this system effectively and induce or overcome drug resistance.

**Fig. 4:**
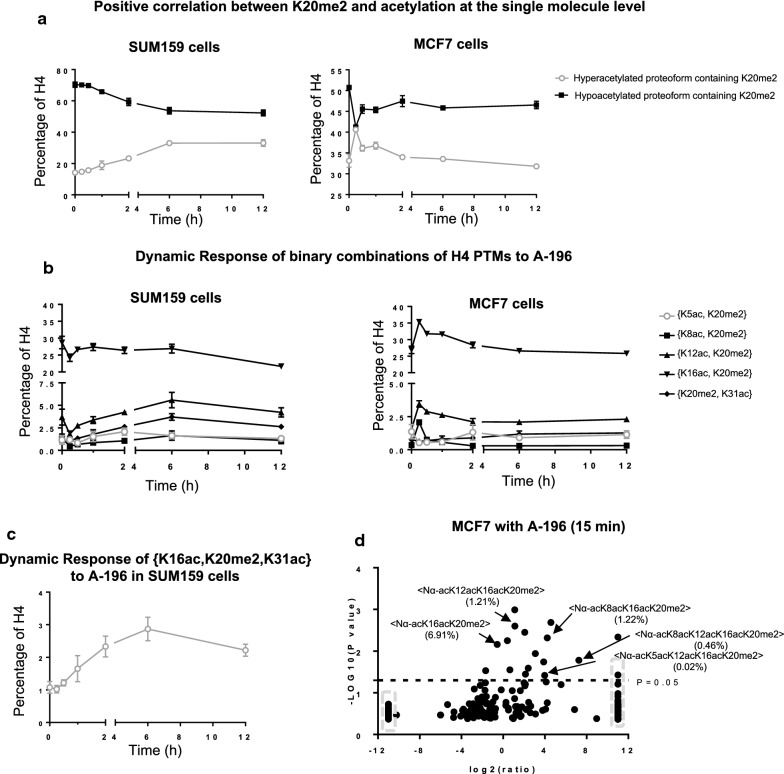
H4K20me2 and H4 acetylation are positively correlated on the single molecule level. **a** Hyperacetylated proteoforms with K20me2 increase, while hypoacetylated proteoforms with K20me2 decrease upon SUV4-20 inhibition in both cell lines. **b** The effects of SUV4-20 inhibition on binary combinations containing K20me2 and H4 acetylation in both cell lines. **c** The ternary combination of {K16ac, K20me2, K31ac} is increased in SUM159 cells upon SUV4-20 inhibition. **d** Volcano plot of proteoform changes in MCF7 cells after 15 min A-196 application. Data points in the gray square indicate infinity fold change. Error bars in **a**, **b**, **c** represent standard error from three biological replicates

Counter to expectations based on the anti-correlation in discrete abundance between H4{K20me2} and acetylation, H4K20me2 and H4 acetylation are positively correlated at the single molecule level in both cell lines (Fig. 4a). That is the single molecule co-occurrence of these PTMs is dramatically increasing. Combinations containing both H4{K20me2} and H4 acetylation primarily increase in abundance in both cell lines:

In SUM159 cells, all binary combinations containing K20me2 and H4 acetylation (except H4{K16ac, K20me2}) increase and reach a plateau after 2–6-h treatment (Fig. 4b, left panel). H4{K16ac, K20me2} decreases quickly (15 min), recovers to the basal level after 6 h treatment, and then decreases again during 6–12-h treatment period.

In MCF7 cells, all binary combinations of K20me2 with acetylation (except H4{K5ac, K20me2}) increase rapidly in about 15 min and then revert to basal levels within an hour or two (Fig. 4b, right panel). It is only after this reversion of acetyl-dimethyl proteoforms, in the 2–6 h time frame that the majority of the slight decrease in discrete H4{K20me2} occurs.

Further analysis of this phenomenon shows that ternary combinations containing K20me2 and any two acetylations are either increased or not altered in these two cell lines. Even, H4{K16ac, K20me2} with any additional acetylation (except Nα-ac) to form a ternary combination in SUM159 cells, i.e., H4{Kxxac, K16acK, 20me2}, is either increased or not changed after 12-h treatment (Additional file 1: Fig. S3A). For example, the ternary combination H4{K16ac, K20me2, K31ac} continuously increases post 12 h SUV4-20 inhibition (Fig. 4c, Additional file 1: S3A). In MCF7 cells, H4{K8ac, K12ac, K20me2}, {K8ac, K16ac, K20me2} and {K12ac, K16ac, K20me2} are all statistically significantly increased in 15 min after treatment (Additional file 1: Figure S3B, right panel).

Proteoforms containing K20me2 and multiple acetylations are increased slowly upon SUV4-20 inhibition in SUM159 cells (Fig. 4a, left panel). Many of the changes observed at the binary combination level (which categorically ignores the PTM occupancy of other sites) are due to more extensively acetylated proteoforms (Additional file 1: Fig. S3C).

Differently from the SUM159 dynamic, hyperacetylated proteoforms with K20me2 are increased quickly upon SUV4-20 inhibition in MCF7 cells (*p* < 0.05), while hypoacetylated ones decrease rapidly and significantly (*p* < 0.0001) (Fig. 4a, right panel). Although there is no significant change to discrete H4{K20me2} for at least 2 h, at 15 min post A-196 application the proteoforms H4<Nα-acK16acK20me2>, H4<Nα-acK12acK16acK20me2> and H4<Nα-acK8acK12acK16acK20me2> are statistically significantly increased, contributing to an overall 8.6% points increase in H4{K20me2} if considered in isolation; however, this is compensated for by the loss of H4<Nα-acK20me2> (Fig. 4d, Additional file 1: S3D; Table S3). The divergent behavior of hypo- and hyperacetylated proteoforms nearly exactly counterbalance each other and explain the lack of change in discrete H4{K20me2} in MCF7 cells.

**Fig. 5:**
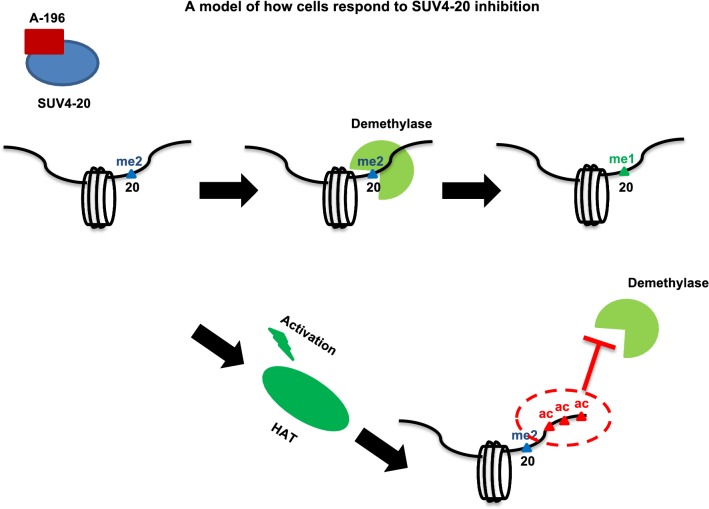
A model for SUV4-20 inhibition Upon SUV4-20 inhibition, K20me2 is demethylated to K20me1 if the molecule is not acetylated (or lightly acetylated). Meanwhile, SUV4-20 inhibition may also activate HAT activity through unknown mechanisms. This increases proteoforms containing K20me2 and multiple acetylations. The presence of multiple acetylations protects K20me2 from being demethylated

The strong anti-correlation of discrete abundance and positive correlation at the single molecule level suggests an evolved compensatory mechanism by which acetylation protects methylation (Fig. 5). The increased abundance of nearly all proteoforms containing K20me2 and multiple acetylations is in stark contrast to the naïve inference that because H4{K20me2} is decreasing, all H4{K20me2} containing species will decrease. In summary, these findings show that (1) not all proteoforms containing H4K20me2 are equally decreased upon SUV4-20 inhibition; (2) some proteoforms containing K20me2 are modified to become highly acetylated proteoforms post-SUV4-20 inhibition; (3) proteoforms containing H4{K20me2} in conjunction with multiple acetylations are resistant to the decreases in H4{K20me2} most directly associated with SUV4-20 inhibition. This suggests that the presence of H4 acetylation prevents the demethylation of H4K20me2. Furthermore, the cellular response that creates these hyperacetylated and dimethylated proteoforms appears to be an evolved mechanism to protect K20me2 and maintain epigenetic state.

To test this hypothesis, we next ask if SUM159 cells could be made resistant to SUV4-20 inhibition by increasing the abundance of hyperacetylated proteoforms containing K20me2 prior to application of A-196? Inversely, can the resistance of MCF7 to SUV4-20 inhibition be diminished by hindering or postponing the increasing of acetylation?

### HDACi pretreatment-mediated H4 acetylations protect K20me2

**Fig. 6:**
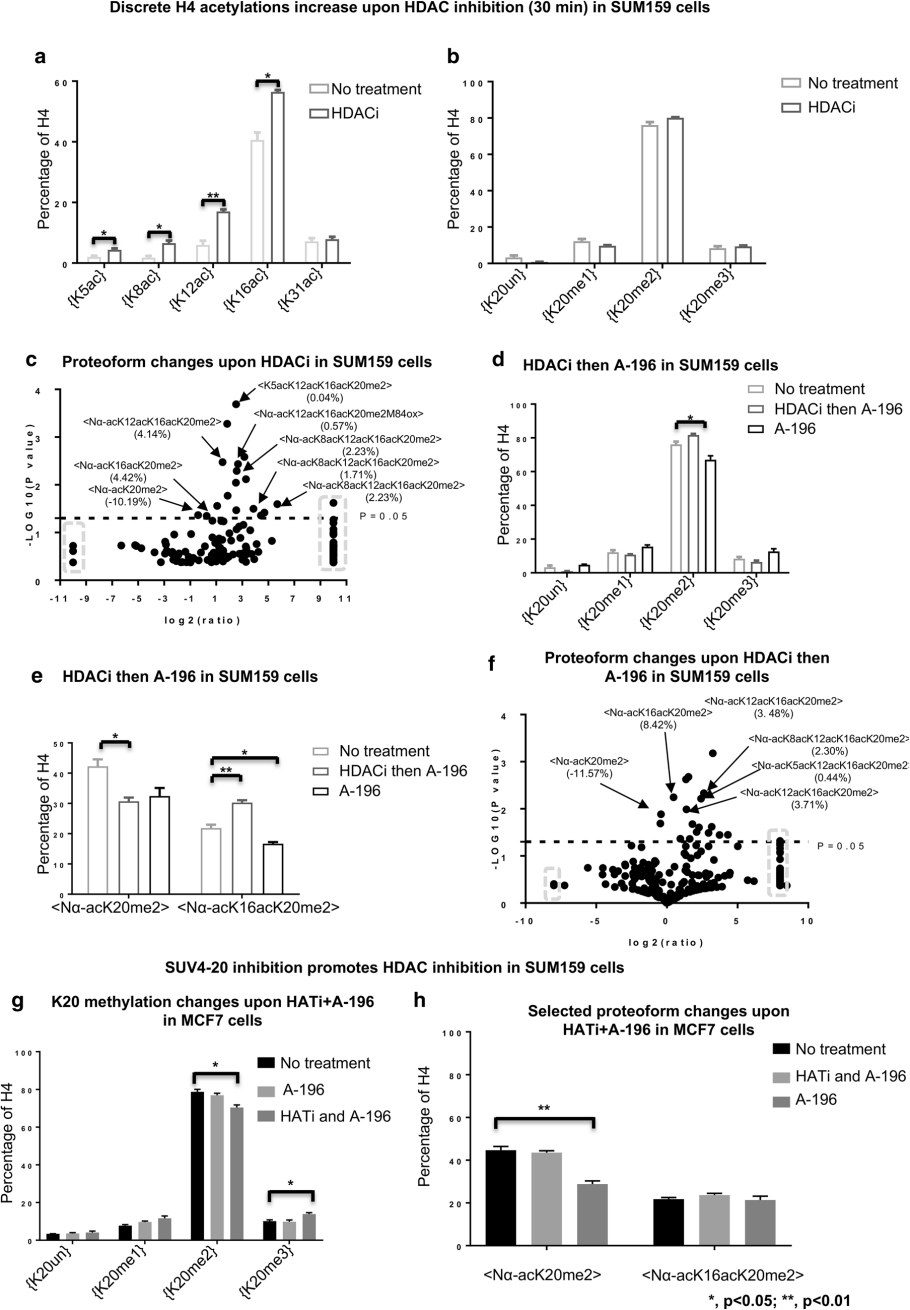
HDAC inhibition diminishes the effects of SUV4-20 inhibition in typically prone SUM159 cells, while HATi facilitates SUV4-20 inhibition in typically resistant MCF7 cells. **a** Discrete H4 acetylations are increased rapidly upon HDAC inhibition. **b** Relative abundance of H4{K20me2} is not decreased in HDACi pretreated SUM159 cells. **c** Volcano plot of changes in proteoform abundance in SUM159 cells upon HDAC inhibition. Data points in the gray dashed squares indicate infinity fold change. **d** HDACi diminishes the effects of SUV4-20 inhibition on discrete H4{K20me2}. **e** The effect of HDACi then SUV4-20 inhibition on the abundance of <Nα-acK20me2> and <Nα-acK16acK20me2> in SUM159 cells. **f** Proteoform changes in response to the presence of HDACi then SUV4-20 inhibition in SUM159 cells. Data points in the gray square indicate infinity fold change. HATi facilitates SUV4-20 inhibition. **g** The effect of HATi then SUV4-20 inhibition on discrete H4K20 methylation states, as well as **h** two proteoforms (<Nα-acK20me2> and <Nα-acK16acK20me2>). *p < 0.05; **p < 0.01

The application of the HDAC inhibitor sodium butyrate to SUM159 cells increases proteoforms containing K20me2 and multiple acetylations; precisely the proteoforms that we hypothesize may protect K20me2 from the effects of SUV4-20 inhibition. As with the inhibition of SUV4-20, the application of the HDACi also results in rapid changes to histone H4 acetylations within 30 min (Fig. 6a, Additional file 1: S4A). Many of these changes are superficially similar to the effects of SUV4-20 inhibition, but substantially different on close inspection. Acetylation increases at all the same sites in similar proportions and in a similar time frame; however, no change to the discrete H4{K20me2} is observed (Fig. 6b, Additional file 1: S4B). At the proteoform level <Nα-acK20me2> decreases as with SUV4-20 inhibition, but this is due to enhanced acetylation of proteoforms containing H4{K20me2} (Fig. 6c; Additional file 1: Table S9). For example, <Nα-acK16acK20me2> is increased by 4.5% points (from 26.3 to 21.8%) upon HDACi, but this proteoform is actually significantly decreased upon SUV4-20 inhibition in SUM159 cells (Additional file 1: Fig. S4C). Central to testing the hypothesis, hyperacetylated proteoforms such as <Nα-acK12acK16acK20me2> and <Nα-acK8acK12acK16acK20me2> are increased significantly (Fig. 6c).

Confirming the hypothesis that acetylation protects H4K20me2 on the single molecule level, pretreatment with the HDAC inhibitor (sodium butyrate) nearly abolishes the effects of SUV4-20 inhibition on H4K20me2 in SUM159 cells. Untreated SUM159 cells incubated with SUV4-20 inhibitor for 2 h results in a 9.2% points decrease of discrete H4{K20me2} (from 76.2 to 67.0%) (Fig. 2b). In cells pretreated with HDAC inhibitor, the discrete H4{K20me2} does not decrease upon SUV4-20 inhibition (Fig. 6d, Additional file 1: S4B). At the proteoform level, <Nα-acK20me2> is decreased by 11.6% points (42.3 to 30.7%), while <Nα-acK16acK20me2>, <Nα-acK12acK16acK20me2> and <Nα-acK8acK12acK16acK20me2> are in total increased by 14.4% points (from 24.6 to 39.0%) (Fig. 6e, f; Additional file 1: Table S10). This confirms the inhibitory effects of multiple acetylations against demethylation of K20me2 noted above at the molecular level. The divergent proteoform response is very similar to what we observe in MCF7 cells after 15 min A-196 application. Thus, we have converted the SUM159 response phenotype to an MCF7-like response phenotype that is resistant to the decreases in discrete H4{K20me2} upon SUV4-20 inhibition. Thus, the outcome is remarkably consistent with our specific proteoform-level hypothesis. In addition, we also find that pretreatment with SUV4-20 inhibitor enhances the effects on discrete H4 acetylations of HDAC inhibition (Additional file 1: Fig. S4D, E). Thus, the epigenetic state of cells is differentially modulated by the application of treatment in different orders.

### HATi enhances the effects of A-196 on H4K20me2 in MCF7 cells

MCF7 cells exhibit a more prone, SUM159-like, molecular response phenotype to SUV4-20 inhibition in the presence of HAT inhibitor, further confirming the hypothesis that the immediate-early increase in HAT activity protects H4{K20me2} levels. To test our hypothesis that the resistance of MCF7 cells is due to the quick activation of HATs, a HAT inhibitor (CTK7A), which specifically targets p300 and PCAF, was employed to hinder or postpone HAT activation in MCF7 cells [32]. Unlike the application of A-196 alone to MCF7, application of both HATi and A-196 results in significantly decreased discrete H4{K20me2} (from 71.18 to 64.99%) (*p* < 0.05) (Fig. 6g). H4{K12ac} and {K16ac} are slightly increased instead of being decreased upon HATi application, while discrete K20 methylation states are not affected by HAT inhibition alone (Additional file 1: Fig. S5A, B), indicating that the HAT activity that is modulated to achieve the hypothesized effect is a dynamic activation of HAT activity. In MCF7 cells, discrete H4{K20me2} is not affected by A-196 alone post 2 h, while, at the same time, <Nα-acK20me2> is only slightly decreased by 1.1% points (from 44.6 to 43.5%) (Fig. 6h). In contrast, co-application of HATi + A-196 causes a 15.8% point decrease of <Nα-acK20me2> from 44.6 to 28.8% (*p* < 0.01), while <Nα-acK16acK20me2> is not affected (Fig. 6h; Additional file 1: Table S11). Therefore, confirming our hypothesis, HATi facilitates the discrete effects on H4{K20me2} from SUV4-20 inhibition in MCF7 cells. In contrast, A-196 alone shows a very limited response. Thus, we have made the previously resistant MCF7 cells prone to A-196 as measured by the discrete H4{K20me2} levels.

In summary, we find that proteoforms containing K20me2 and multiple acetylations are not prone to the demethylation events that dominate upon SUV4-20 inhibition. This has been confirmed by multiple hypothesis validation experiments. This explains why in some contexts H4{K20me2} is decreased slightly as these hyperacetylated proteoforms increase. Additionally, it is clear that dynamic HAT activation contributes to the resistance of MCF7 cells to the discrete effects of SUV4-20 inhibition by acetylating molecules with K20me2 present and thus protecting the K20me2 from demethylase activity.

## Discussion

The dynamics of discrete H4 PTMs and the cross-talk between H4 PTMs on single molecules are scarcely understood. Here, we apply quantitative top-down proteomics with unprecedented reproducibly to analyze the dynamics of histone H4 with single molecule connectivity between PTMs in two breast cancer cell lines upon epigenetic therapy. With this method, we can measure histone PTMs individually or concurrently on the same molecule. Our data provides new insights into the dynamic interplay between H4{K20me2} and H4 acetylation states.

### Proteoforms, not discrete PTMs, are associated with diverse biological function

We posit that proteoforms instead of discrete PTMs are the signals in many biological events. Dysregulation of PTMs have been considered as candidate biomarkers for various cancer types, including H4K16ac and H4K20me3 [33]. However, many such correlations are hard to reconcile with quantitative analysis of discrete PTM abundance. For example, we find that in breast cancer cell lines more than 40% of H4 molecules have H4K16ac. Despite previous associations, it is clear that the complete loss of all proteoforms containing H4K16ac is not associated with carcinogenesis. However, K16ac depletion in the context of specific proteoforms may be a crucial component. Likewise, H4K20me2 is not only enriched in euchromatin regions [34], but also in heterochromatin regions [35]. How does one discrete PTM serve as a hallmark for both euchromatin and heterochromatin? Our data reveals that more than 70% of H4 molecules have K20me2. It is hard to imagine that discrete H4{K20me2} in isolation from other PTMs carries a unique, much less a gene activating signal with an abundance > 70%. Note that due to the presence of two copies of H4 per nucleosome, the nucleosomal presence is likely approaching 100%. It appears necessary that K20me2 functions in conjunction with other PTMs in different chromatin regions to convey multiple signals of diverse meaning. Thus, we submit that taxonomically classifying the role of discrete histone PTM is insufficient to decipher chromatin regulation.

The single molecule-level response to epigenetic inhibitors is distinct from observed discrete PTMs changes, despite the ‘ensemble’ versus ‘ensemble average’ relationship. Here, we find that not all proteoforms containing K20me2 respond to SUV4-20 inhibition equally. Two proteoforms: H4<Nα-acK20me2> and H4<Nα-acK16acK20me2> are decreased in SUM159 cells, while only H4<Nα-acK20me2> is slightly decreased in MCF7 cells due to SUV4-20 inhibition. <Nα-acK20me2> is the most abundant and accounts for ~ 40% of H4 in both cell lines. It is not clear why this proteoform is more sensitive to SUV4-20 inhibition in SUM159 cells; however, previous work shows that the K20 methyltransferase (SUV4-20) prefers modifying newly synthesized H4 [10]. This proteoform may represent histones recently incorporated into chromatin and are more accessible for demethylase activity, or the absence of neighboring acetylation may make it prone to demethylation. All this evidence suggests that proteoforms, i.e., combinations of histone PTMs, not discrete PTMs, play a central role in chromatin regulation.

### Histone PTMs are regulated via dynamic equilibrium processes

H4K20me2 is dynamically regulated. In both cell lines, H4K20me2 is affected immediately at the proteoform level upon SUV4-20 inhibition. It has been shown that the turnover rate of K20me2 is very slow in vivo [10]. The steady-state half-life of K20me2 is more than 24 h [36]. However, the dynamics of K20me2 upon perturbation, as distinct from the integrated steady-state turnover, have not been previously characterized in any context. We observe that the net loss of K20me2 is about 9.2% points (from 76.2 to 67.0%) after 2 h of SUV4-20 inhibition in SUM159 cells. In MCF7 cells, H4{K20me2} is not affected significantly after 12 h SUV4-20 inhibition. However, proteoforms containing K20me2 are affected immediately by SUV4-20 inhibition. H4K20me2 also recovers immediately in SUM159 cells after removal of the inhibitor from the media. After removal of the SUV4-20 inhibitor, discrete H4{K20me2} levels fully recover in 15 min. The two proteoforms, H4<Nα-acK20me2> and H4<Nα-acK16acK20me2>, that contributed the most to the loss of K20me2, also fully recover. It is important to note that dynamic regulation does not necessitate high sustained steady-state turnover if mechanisms of pausing are present. That is under steady-state conditions enzymatic activity may approach zero but perturbations may trigger transient spikes in enzymatic activity. All this evidence implies that histone PTMs are regulated in a very dynamic manner.

### H4K20me3 responds to SUV4-20 differently, compared to H4K20me2

Our results contradict previous studies showing decreases in H4{K20me3} upon SUV4-20 inhibition. Neither discrete H4{K20me3} nor H4{K20me3} containing proteoforms decrease upon SUV4-20 inhibition at these timesscales, despite extraordinarily rapid decreases in H4{K20me2}. In previous work, K20me3 is significantly decreased both in vivo and in vitro in the presence of A-196 on the 48 h timescale [29]. However, our data suggest that K20me3 does not decrease for at least at 12 h post-A196 application. Thus, longer-term changes in K20me3 may be due to downstream and not direct effects.

These results are incompatible with the prevailing perception that SUV4-20 is the sole enzyme responsible for H4{K20me3}. The work of Bromberg et al. strongly supports A-196 inhibiting both SUV4-20H1 and SUV4-20H2. It is thus likely that another enzyme is the ‘writer’ forH4K20me3. Indeed, other literature supports this hypothesis. For example, Wu et al. [27] have shown that SUV4-20 does not tri-methylate K20 in vitro. It has also been suggested that the SET and MYND Domain (SMYD) proteins have the potential to modify K20me3 [37].

### The synergy between H4 methylation and acetylation

Surprisingly, SUV4-20 inhibition not only decreases H4K20me2, but also increases H4 acetylation states at the discrete, binary combination and proteoform levels. H4{K5ac}, {K8ac} and {K31ac} increase significantly and gradually in SUM159 cells, while H4{K8ac}, {K12ac} and {K16ac} dramatically, quickly and transiently increase in MCF7 cells. Note that in the recent work by Bromberg et al., in which they conclude that there is no change in acetylation status upon SUV4-20 inhibition, they did not measure the acetylations that change most substantially. The acetylation sites that they monitored exhibit only modest fold changes that are near impossible to observe by traditional methods. How the decrease of H4{K20me2} causes the change in H4 acetylation states is not understood; however, it may be an evolved compensatory mechanism where cells attempt to recover from SUV4-20 inhibition by increasing H4 acetylation levels and protecting H4{K20me2} from demethylation. Indeed, K20me2 and H4 acetylation are positively correlated at the single molecule level. We find that hyperacetylated proteoforms with K20me2 are specifically increased upon SUV4-20 inhibition. In agreement, previous studies have shown that eukaryotes have evolved mechanisms to survive natural epigenetic inhibitors and this impacts the efficacy of epigenetic inhibitors as cancer therapeutics [38]. Such evolved mechanisms of resistance to epigenetic changes also explain how histone PTMs may both be dynamically regulated and stable over very long time-frames.

We propose a data-driven model describing how SUV4-20 inhibition only affects specific proteoforms (Fig. 5). Upon SUV4-20 inhibition, hypoacetylated proteoforms with K20me2 are demethylated to K20me1. Meanwhile, HATs are activated through unknown mechanisms, which cause an increase of specific proteoforms containing K20me2 and hyperacetylations. These proteoforms are resistant to demethylase activity. How acetylation protects K20me2 is still not clear. However, the most likely mechanism is that each proteoform is a unique substrate. The demethylase simply does not recognize hyperacetylated and K20 dimethylated histone H4 and is thus enzymatically inactive against such proteoforms. Highly acetylated chromatin regions may also serve as binding sites for regulatory proteins containing bromodomains. These proteins may compete with demethylases to bind to this region, thus preventing the loss of K20me2. Overall, our data suggest strong dependencies between K20me2 and H4 acetylation states and that this interplay is important to maintaining epigenetic state.

H4K20me2 is protected by the coexistence of multiple H4 acetylations on the same molecule. We find that SUV4-20 inhibition causes the decrease of only hypoacetylated proteoforms-{K20me2}. This difference in H4K20me2 behavior between hypoacetylated and hyperacetylated H4{K20me2} containing proteoforms is further corroborated by the fact that SUV4-20 inhibition does not affect discrete H4{K20me2} levels after transient HDAC inhibition, as shown in Fig. 6b. This indicates that increased hyperacetylated proteoforms-{K20me2} make SUM159 cells resistant to SUV4-20 inhibition, in agreement with our hypothetical model (Fig. 5). The quick increase in hyperacetylated-{K20me2} due to HAT activation after SUV4-20 inhibition is proposed to contribute to the protection of K20me2 according to our hypothetical model (Fig. 5). Therefore, we tested this part of our model by use of a histone acetyltransferase inhibitor (HATi) in conjunction with A-196. Upon HATi and A-196 application, H4{K20me2} is statistically significantly decreased in MCF7 cells. This suggests that inhibition of HAT makes MCF7 cells prone to SUV4-20 inhibition, confirming our hypothesis. Surprisingly, hyperacetylated proteoforms with K20me2 are still increased upon application of either HATi alone or HATi and A-196 (Additional file 1: Fig. S5C). Notably, the HAT inhibitor we employ here only targets p300 and PCAF and does not obliterate all activity [32]. Furthermore, other HATs may be involved in the acetylation-dependent protection of K20me2. These caveats explain why discrete H4{K20me2} is only decreased by ~ 8% points (from 78.7 to 70.4%) upon HATi and A-196 application compared to 15.8% points decrease (from 76.2 to 60.4%) in SUM159 cells upon A-196 alone, and why hyperacetylated proteoforms with K20me2 and the discrete acetylations are still increased.

### Summary of our impact

The results presented here demonstrate that histone PTMs and chromatin are dynamically regulated and that these short timescale changes occur on specific proteoforms with specificity toward preexisting PTMs. We have shown that histone PTMs are regulated in an unprecedentedly dynamic manner and exhibit changes within minutes of perturbation. This indicates that DNA-dependent processes can be modulated in minutes and perhaps even in seconds. This idea is supported by previous results that gene transcript levels are altered significantly by HDAC inhibition within 30 min [31]. The synergy observed here among histone PTMs (e.g., H4K20me2 and H4 acetylations) suggests that proteoforms, and not single PTMs, are the true regulators for many fundamental biological processes. Without the sort of data we present here it is difficult, if not impossible, to fully understand or predict how histone PTMs regulate biological processes. Additionally, any single histone PTM may be modified by multiple enzymes. For examples, H4K16ac can be modified by either MOF or Tip60 [39, 40]. It may appear that these enzymes are redundant; however, our data suggests that enzymes may have different proteoform-level specificity. These ‘writers’ may only function on certain proteoforms, although they are associated with the same discrete PTMs. This hypothesis will be further confirmed by future in vitro proteoform-level enzymology assays currently in progress (data not shown). Overall, our current work provides new insight into the importance of understanding fundamental chromatin biochemistry through the lens of quantitative measurement of histone proteoforms and the combinations of PTMs that they harbor at the single molecule level.

### Final conclusion

Overall, we find that H4{K20me2} is decreased within 15 min post-SUV4-20 inhibition. As expected, K20me1 concomitantly increases. Specific proteoforms are affected rapidly and dramatically. H4{K20me3} is not affected after 12 h of SUV4-20 inhibition, despite SUV4-20 being generally, if not universally, accepted as the exclusive enzyme for the writing of H4{K20me3}. Inhibition of SUV4-20 results in global increases of H4 acetylation states, most dramatically at K5 and K31 in SUM159 cells, and at K8, K12 and K16 in MCF7 cells. Our results suggest a strong bidirectional correlation between K20 methylation status and acetylation states. This model is supported by the evidence that increased levels of acetylation upon HDACi pretreatment prevent the loss of K20me2 upon subsequent SUV4-20 inhibition. Additionally, inactivation of HATs diminishes the resistance of MCF7 cells to SUV4-20 inhibition, further corroborating our overarching hypothesis. Overall, the network of histone PTMs we observe here is indicative of a much more dynamic, nuanced and complex chromatin regulatory system than the current knowledge generally attributes.

## Methods

### Tissue culture

SUM159 and MCF7 cells were maintained according to a protocol adapted from the American Type Culture Collection (Additional file 1).

### SUV4-20 inhibition

SUM159 and MCF7 cells were treated with 1 µM SUV4-20 inhibitor (A-196). Three dishes per time point were harvested at: 0, 15, 30 min, 1, 2, 6 and 12 h.

### Combination of HDACi with SUV4-20 inhibition

SUM159 cells were treated with 5 mM sodium butyrate for 30 min. At which point, A-196 (1 µM) was added and incubated for another 2 h. Separately, in an experiment reversing this order, cells were pretreated with A-196 for 2 h and sodium butyrate (5 mM) was added to the media and incubated for another 30 min.

### Combination of HATi with SUV4-20 inhibition

MCF7 cells were treated with 100 µM HATi (CTK7A, Calbiochem) for 4 h, followed by addition of sufficient stock to give 100 µM HATi + 1 µM A-196 for another 2 h.

### Histones extraction and purification

Histones were extracted by acid extraction as previously described [41].

### Lc–MS/MS

Online liquid chromatography was performed with a Thermo Scientific Dionex UltiMate 3000 RSLCnano System with a ProFlow Pump block (Additional file 1).

### Data analysis

Data analysis method was adapted [42] and has been further optimized for intact H4 with ETD fragmentation (Additional file 1).

## Additional file

**Additional file 1.** Further description of notation, method details, and additional data. The notation used to describe the various levels of combinations and full proteoforms are discussed in further detail. The details of the methods are described. Substantial additional results tables and figures that are supportive of the central findings but are too extensive for inclusion in the main text are presented.
