# Supplementary material for: The histone H4 proteoform dynamics in response to SUV4-20 inhibition reveals single molecule mechanisms of inhibitor resistance
Source: Epigenetics Chromatin. 2018 Jun 7;11:29. doi: 10.1186/s13072-018-0198-9 (PMC5992683; doi:10.1186/s13072-018-0198-9)
Supplement: Supplementary file 1 — Additional file 1. Further description of notation, method details, and additional data. The notation used to describe the various levels of combinations and full proteoforms are discussed in further detail. The details of the methods are described. Substantial additional results tables and figures that are supportive of the central findings but are too extensive for inclusion in the main text are presented. [file 13072_2018_198_MOESM1_ESM.pdf]

Additional file 1

Notation

We present data of an unconventional level of detail at multiple overlapping and interrelated levels. This necessitates the use of novel nomenclature and notation to adequately describe and sufficiently distinguish different interrelated concepts of molecular identity concisely. The abundance of an individual PTM as conceived in the prevailing literature is herein referred to as a 'discrete PTM', indicating that it is considered 'separately and distinctly' from other PTMs that ambiguously may or may not exist on the same molecule. This is conceptually similar to the manner in which a western blot or bottom-up proteomics measures a PTM. In our analysis, this is actually derived from a marginalization process in which all proteoforms containing this PTM are added and then normalized to 100%. Notationally, this is indicated by curly brackets containing a single Turner style PTM (Turner 2005). For example, 'the discrete PTM H4K20me2' is indicated concisely by simply: H4{K20me2}. This is necessary to distinguish it from the proteoform that contains exclusively dimethylation at lysine 20, indicated by H4<K20me2>, (with all other sites of variable modification unambiguously unmodified). In the same way, discrete binary and ternary combinations are quantitated, which is simply denoted as H4{PTM1, PTM2} and H4{PTM1, PTM2, PTM3}, respectively. The notation H4<PTM1PTM2PTM3> is used to denote a specific proteoform, where all possible histone modification combinations are considered on a single molecule level. For instance, H4<K5acK20me3> represents a proteoform containing exclusively K5ac and K20me3 and definitively no other PTMs on this single H4 molecule. The "<>" notation indicates that all sites not specifically designated are unambiguously

unmodified, while the “{}” notation definitively ignores or marginalizes the PTM occupancy of all sites not specifically designated. It may be helpful to understand that “<>” represents a sparse vector while “{}” represents a set. At times, we also refer to generic PTMs without designation of location. We present data of an unconventional level of detail at multiple overlapping and interrelated levels. This necessitates the use of novel nomenclature and notation to adequately describe and sufficiently distinguish different interrelated concepts of molecular identity concisely. The abundance of an individual PTM as conceived in the prevailing literature is herein referred to as a ‘discrete PTM’, indicating that it is considered ‘separately and distinctly’ from other PTMs that ambiguously may or may not exist on the same molecule. This is conceptually similar to the manner in which a western blot or bottom-up proteomics measures a PTM. In our analysis, this is actually derived from a marginalization process in which all proteoforms containing this PTM are added and then normalized to 100%. Notationally, this is indicated by curly brackets containing a single Turner style PTM (Turner 2005). For example, ‘the discrete PTM H4K20me2’ is indicated concisely by simply: H4{K20me2}. This is necessary to distinguish it from the proteoform that contains exclusively dimethylation at lysine 20, indicated by H4<K20me2>, (with all other sites of variable modification unambiguously unmodified). In the same way, discrete binary and ternary combinations are quantitated, which is simply denoted as H4{PTM1, PTM2} and H4{PTM1, PTM2, PTM3}, respectively. The notation H4<PTM1PTM2PTM3> is used to denote a specific proteoform, where all possible histone modification combinations are considered on a single molecule level. For

instance, H4<K5acK20me3> represents a proteoform containing exclusively K5ac and K20me3 and definitively no other PTMs on this single H4 molecule. The “<>” notation indicates that all sites not specifically designated are unambiguously unmodified, while the “{}” notation definitively ignores or marginalizes the PTM occupancy of all sites not specifically designated. It may be helpful to understand that “<>” represents a sparse vector while “{}” represents a set. At times, we also refer to generic PTMs without designation of location.

Methods and Materials

Tissue culture

SUM159 and MCF7 cells were maintained according to a protocol adapted from the American Type Culture Collection. Cells were grown at 37°C with 5% CO2. SUM159 cells were grown in F-12 Nutrient Mixture (Life Technologies) supplemented with 10% newborn calf serum (NCS), 100 U/ml penicillin and streptomycin, 10 mM HEPES, and 1 µg/ml hydrocortisone. MCF7 cells were maintained in Dulbecco's Modified Eagle Medium (Life Technologies) supplemented with 10% NCS and 100 U/ml penicillin and streptomycin.

LC-MS/MS

Online liquid chromatography was performed with a Thermo Scientific DIONEX UltiMate 3000 RSLCnano System with a ProFlow Pump block. Buffers used were: Buffer A: 98% H2O, 2% ACN, 0.1% FA Buffer B: 2% H2O, 98% ACN, 0.1% FA. A linear gradient from 27% B to 30% B in 70 min was used, followed by a subsequent wash. A single homemade column measuring 15 cm in length, 100 µm ID, and

packed with ZORBAX C3-300SB, 3.5 µm bead size with 300 Å pores was used for all experiments. 1µL of sample was injected which contains approximately 100 ng of histone H4 obtained from offline fractionation. A Nanospray Flex source was used with 1700 Positive Ion Voltage and 320 °C Ion Transfer Tube Temperature. Mass spectrometry was performed with a Thermo Scientific Orbitrap Fusion Lumos operating in positive mode and intact protein mode. For MS1, the orbitrap detector was used with a resolution setting of 60,000 (sufficient for isotopic separation), a scan window of 700-1400 *m/z*, 5.0e5 AGC, 80% RF, 200 ms injection time and 5 microscans. A targeted mass list was used to pick MS1 peaks for fragmentation (752.8843, 753.8195, 754.7546 … 781.8737 *m/z* ± 0.20 *m/z*) that captures all significant MS1 peaks for the 15th charge state of histone H4. An additional 1.0e5 threshold was used and the top 20 most abundant MS1 peaks were selected for MS2. Precursor ions were isolated by the quadrupole with a 1 *m/z* isolation width prior to ETD fragmentation. ETD was carried out with a 14 ms ETD Reaction Time, 5.0e5 ETD Reagent Target and 200 ms ETD Reagent Injection Time. The Orbitrap was used for MS2 analysis with Auto: *m/z* normal Scan Range mode, 60,000 resolution setting, an AGC target of 5.0e5, 3 microscans and Profile data type.

Data Analysis

Data analysis method was adapted (DiMaggio et al. 2009), and has been further optimized for intact H4 with ETD fragmentation. Briefly, this software takes into consideration both MS1 and MS2 spectra, over the course of a chromatogram, to both characterize and quantify specific proteoforms. The resulting identifications and abundances were normalized to the entire signal resulting in relative

quantitation, such that results are reported as the % of the total histone H4 in the cell.

Table S1: Proteoform-level comparison between SUM159 and MCF7 cells.

Proteoforms	SUM159	MCF7	Difference	Fold change	P value
<Nα-acK8acK16acK20me2>	0.68%	0.06%	-0.62%	0.08	0.00052
<Nα-acK12acK16acK20me2K31ac>	0.56%	0.25%	-0.30%	0.45	0.01061
<Nα-acK16acK20me1>	3.35%	1.86%	-1.49%	0.55	0.02488
<K5acK16acK20me3>	0.05%	0.18%	0.13%	3.70	0.03332
<Nα-acK12acK16acK20me1>	0.44%	0.03%	-0.41%	0.06	0.04180

Table S2: Proteoform changes in response to SUV4-20 inhibitor (12 h) in SUM159 cells.

Proteoforms	SUM159	A196 12 h	Difference	Fold change	P value
<Nα-acK12acK16ac>	0.02%	0.35%	0.33%	0.94	5.5E-05
<Nα-acK12acK16acK20me1K31acM84ox>	0.01%	0.27%	0.26%	0.98	3.5E-04
<Nα-acK20me1M84ox>	0.60%	3.97%	3.38%	0.85	3.8E-04
<Nα-acK8acK12acK16acK20me2M84ox>	0.01%	0.08%	0.08%	0.93	3.8E-04
<Nα-acK20me3K31acM84ox>	0.07%	0.31%	0.24%	0.78	1.3E-03
<Nα-acM84ox>	0.18%	2.38%	2.20%	0.92	2.8E-03
<Nα-acK16acK20me2>	21.84%	9.26%	-12.58%	-1.36	3.3E-03
<S1phR3me1K5acK12acK16acK20me1M84ox>	0.00%	0.01%	0.01%	1.00	3.5E-03
<S1phK8acK16acK20me2K31acM84ox>	0.00%	0.00%	0.00%	1.00	3.5E-03
<Nα-acK20me2>	42.26%	23.40%	-18.86%	-0.81	3.7E-03
<Nα-acK20me2M84ox>	4.30%	13.05%	8.74%	0.67	3.9E-03
<Nα-acK16acM84ox>	0.02%	0.90%	0.88%	0.98	4.5E-03
<Nα-acK16acK20me2M84ox>	1.88%	6.56%	4.68%	0.71	5.6E-03
<Nα-acK12acK16acK20me2M84ox>	0.11%	0.86%	0.75%	0.88	6.3E-03
<Nα-acK16acK20me1M84ox>	0.26%	2.23%	1.97%	0.88	7.6E-03
<Nα-acK8acK16acK20me2>	0.64%	0.01%	-0.62%	-44.50	1.2E-02
<Nα-acK12acK16acK20me2K31ac>	0.51%	0.39%	-0.12%	-0.31	1.2E-02
<Nα-acK8acK12acK16acK20me3K31acM84ox>	0.01%	0.06%	0.05%	0.87	1.3E-02
<Nα-acK8acK12acK16acK20me1K31acM84ox>	0.00%	0.04%	0.04%	0.98	1.3E-02
<Nα-acK12acK16acK20me2K31acM84ox>	0.00%	0.16%	0.16%	1.00	1.4E-02
<Nα-acK12acK16acK20me1M84ox>	0.01%	0.31%	0.30%	0.98	1.5E-02

<Nα-acK12acK16acM84ox>	0.00%	0.18%	0.18%	0.99	1.5E-02
<S1phK8acK12acK20me3K31ac>	0.00%	0.02%	0.02%	1.00	1.7E-02
<K5acK16acK20me3>	0.06%	0.01%	-0.05%	-4.25	1.9E-02
<Nα-acK20me3M84ox>	0.21%	0.74%	0.53%	0.71	2.1E-02
<S1phR3me1K5acK20me2>	0.00%	0.00%	0.00%	1.00	2.5E-02
<Nα-acS1phK5ac>	0.00%	0.03%	0.03%	1.00	2.9E-02
<Nα-acK5acK8acK20me3>	0.00%	0.08%	0.08%	1.00	2.9E-02
<Nα-acK5acK8acK12acK16acK31ac>	0.00%	0.18%	0.18%	1.00	3.0E-02
<Nα-acK12acK16acK20me3K31acM84ox>	0.08%	0.42%	0.34%	0.81	3.2E-02
<Nα-acK5acK8acK16ac>	0.00%	0.08%	0.08%	1.00	3.3E-02
<K5acK16acK20me2K31ac>	0.00%	0.01%	0.01%	0.97	3.5E-02
<Nα-acK20me3>	1.71%	0.52%	-1.19%	-2.27	3.6E-02
<K5acK12acK16acK20me1M84ox>	0.00%	0.01%	0.00%	0.76	3.9E-02
<Nα-acS1phK5acK8acK16ac>	0.00%	0.08%	0.08%	1.00	4.3E-02
<Nα-acK16acK20me3M84ox>	0.06%	0.32%	0.25%	0.80	4.4E-02
<Nα-acS1phR3me1K5acK12acK16acK31acM84ox>	0.00%	0.00%	0.00%	∞	4.5E-02
<K5acK12acK16acK20me2>	0.01%	0.04%	0.03%	0.78	4.6E-02
<K5acK8acK12acK16acK20me3K31ac>	0.00%	0.09%	0.08%	0.97	4.7E-02

Table S3: Proteoform changes in response to SUV4-20 inhibitor (15 min) in MCF7 cells.

Proteoforms	MCF7	A196 15 min	Difference	Fold change	P value
<Nα-acK16acK20me1>	1.81%	3.92%	2.11%	2.16	0.00102
<Nα-K12acK16acK20me1>	0.03%	0.63%	0.60%	23.89	0.00206
<Nα-K12acK16acK20me2>	1.02%	2.23%	1.21%	2.19	0.00248
<K5acK8acK12acK16acK20me3K31ac>	0.00%	0.01%	0.01%	4.19	0.00356
<Nα-K8acK12acK16acK31acM84ox>	0.00%	0.00%	0.00%	∞	0.00456
<Nα-K8acK16acK20me2>	0.07%	1.29%	1.22%	18.81	0.00474
<Nα-K16acK20me2>	21.75%	28.66%	6.91%	1.32	0.00564
<Nα->	2.69%	1.83%	-0.86%	0.68	0.00697
<Nα-K8acK12acK16acK20me3K31ac>	0.02%	0.18%	0.16%	8.50	0.01157
<Nα-K8acK12acK16acK20me2>	0.00%	0.47%	0.46%	152.99	0.01657
<K8acK16acK20me3K31ac>	0.00%	0.00%	0.00%	14.81	0.01806
<Nα-K8acK12acK16acK20me1>	0.01%	0.04%	0.03%	5.27	0.02574
<K8acK16acK20me3>	0.11%	0.18%	0.06%	1.56	0.02803
<Nα-K5acK8acK12acK16acK20me3M84ox>	0.00%	0.00%	0.00%	0.31	0.02960
<K5acK16acK20me3K31ac>	0.03%	0.12%	0.10%	4.39	0.03530
<S1phK5acK16acK20me2>	0.00%	0.00%	0.00%	∞	0.03700

<Nα-K5acK12acK16acK20me2>	0.00%	0.02%	0.02%	15.77	0.03764

Table S4: Proteoform changes in response to SUV4-20 inhibitor (12 h) in MCF7 cells

Proteoforms	MCF7	A196 12 h	Difference	Fold change	P value
<Nα-acK12acK16acK20me1>	0.03%	0.41%	0.38%	15.56	0.00012
<Nα-acK16acK20me1>	1.81%	4.43%	2.62%	2.44	0.00085
<Nα-acK5acK8acK12acK16acK20me3M84ox>	0.00%	0.00%	0.00%	0.11	0.00386
<Nα-acK12acK16acK20me1K31ac>	0.03%	0.18%	0.15%	7.03	0.00410
<Nα-acK20me1>	4.25%	7.35%	3.10%	1.73	0.00650
<Nα-ac>	2.69%	1.87%	-0.82%	0.70	0.01494
<S1phK5acK8acK16acK20me3K31acM84ox>	0.00%	0.00%	0.00%	0.00	0.01573
<Nα-acK16acK20me2K31ac>	0.32%	0.68%	0.36%	2.14	0.01663
<K5acK8acK12acK16acK20me1>	0.00%	0.00%	0.00%	9.85	0.02193
<K5acK12acK16acK31acM84ox>	0.00%	0.00%	0.00%	0.21	0.02335
<R3me1K12acK16acK20me2K31acM84ox>	0.00%	0.00%	0.00%	7.24	0.02782
<K20me2M84ox>	0.00%	0.00%	0.00%	3.32	0.03638
<Nα-acK12acK16acK20me2>	1.02%	1.59%	0.57%	1.56	0.04854

Table S5: Proteoform changes in response to SUV4-20 inhibitor in SUM159 cells (2 h)

Proteoforms	SUM159	A196 2 h	Difference	Fold change	P value
<S1phK8acK12acK20me3K31ac>	0.00%	0.02%	0.02%	∞	0.00571
<Nα-acK8acK16acK20me2>	0.64%	0.06%	-0.57%	0.10	0.01305
<K5acK8acK12acK16acK20me2K31ac>	0.00%	0.01%	0.01%	28.07	0.02144
<S1phR3me1K12acK20me2>	0.00%	0.01%	0.01%	∞	0.03015
<Nα-acK16acK20me2>	21.84%	16.70%	-5.14%	0.76	0.03114
<Nα-acS1phR3me1K5acK8acK12acK16acK20me2K31ac>	0.00%	0.03%	0.03%	18.58	0.03141
<Nα-acK12acK16acK20me3K31acM84ox>	0.08%	0.24%	0.16%	3.07	0.03575
<Nα-acK5acK12acK20me3>	0.00%	0.03%	0.03%	∞	0.03732
<S1phR3me1K5acK12acK16acK20me1M84ox>	0.00%	0.01%	0.01%	∞	0.03924
<K5acK12acK16acK20me1K31ac>	0.01%	0.04%	0.03%	5.60	0.04097
<Nα-acK5acK8acK12acK16acK20me3>	0.00%	0.03%	0.03%	∞	0.04665

Table S6: Residual proteoform changes in 15 min after removing SUV4-20 inhibitor in SUM159 cells.

Proteoforms	SUM159	A196 Wash off 15 min	Difference	Fold change	P value
<S1phR3me1K20me2>	0.01%	0.00%	-0.01%	0.12	0.00658
<Nα-acS1phK5acK8acK12acK16ac>	0.00%	0.00%	0.00%	20.24	0.01828
<K8acK12acK16acK20me3K31acM84ox>	0.00%	0.00%	0.00%	∞	0.01923
<Nα-acK12acK16acK20me2K31ac>	0.51%	0.22%	-0.29%	0.42	0.02236
<K8acK12acK16acK20me2K31acM84ox>	0.00%	0.00%	0.00%	3.92	0.02280

<K8acK20me2>	0.01%	0.07%	0.06%	8.47	0.02561
<Nα-acK16acK20me1>	3.51%	2.37%	-1.14%	0.68	0.02954
<Nα-acK20me2M84ox>	4.30%	9.44%	5.14%	2.19	0.03500
<Nα-acK20me3>	1.71%	0.62%	-1.09%	0.36	0.03555
<S1phR3me1K8acK12acK16acK20me3K31acM84ox>	0.00%	0.00%	0.00%	0.00	0.03603
<Nα-acK16acK20me2M84ox>	1.88%	3.52%	1.64%	1.87	0.03680
<Nα-acK12acK16acK20me1>	0.51%	0.15%	-0.35%	0.30	0.04795

Table S7: Residual proteoform changes in 30 min after removing SUV4-20 inhibitor in SUM159 cells

Proteoforms	SUM159	A196 wash off 30 min	Difference	Fold change	P value
<Nα-acK16acK20me2>	21.84%	27.61%	5.77%	1.26	0.02093
<K8acK20me2M84ox>	0.00%	0.01%	0.01%	∞	0.02444
<Nα-acK12acK20me3>	0.00%	0.01%	0.01%	58.55	0.02569
<K5ac>	0.01%	0.03%	0.02%	2.33	0.03706
<K5acK16acK20me2M84ox>	0.03%	0.10%	0.06%	2.85	0.03911
<K5acK8acK12acK16acK20me2>	0.04%	0.13%	0.09%	3.00	0.04572
<Nα-acK8acK12acK16acK20me3K31acM84ox>	0.01%	0.00%	0.00%	0.35	0.04632
<K5acK16acK20me2>	0.04%	0.11%	0.06%	2.54	0.04865

Table S8: Proteoform changes in response to SUV4-20 inhibitor (15 min) in SUM159 cells.

Proteoforms	SUM159	A196 15 min	Difference	Fold change	P value
<K5acK16acK20me2M84ox>	0.03%	0.13%	0.09%	3.76	0.0055
<S1phR3me1K20me2>	0.01%	0.00%	-0.01%	0.00	0.0056
<K5acK16acK20me2K31ac>	0.00%	0.01%	0.01%	83.48	0.0102
<Nα-acK8acK16acK20me2>	0.64%	0.28%	-0.35%	0.45	0.0243
<Nα-acK20me2M84ox>	4.30%	11.37%	7.07%	2.64	0.0292
<Nα-acK16acK20me2M84ox>	1.88%	4.40%	2.52%	2.34	0.0301
<S1phR3me1K8acK12acK16acK20me3K31acM84ox>	0.00%	0.00%	0.00%	0.04	0.0342
<Nα-acK16acK20me1M84ox>	0.26%	0.79%	0.52%	2.98	0.0400
<Nα-acK16acK20me2>	21.84%	16.79%	-5.05%	0.77	0.0408
<Nα-acK12acK16acK20me1>	0.51%	0.13%	-0.37%	0.26	0.0410
<K5ac>	0.01%	0.03%	0.02%	2.27	0.0427
<Nα-acS1phR3me1K5acK12acK16acK31acM84ox>	0.00%	0.00%	0.00%	0.00	0.0446
<Nα-acK16acK20me3K31acM84ox>	0.33%	0.90%	0.57%	2.75	0.0462
<Nα-acK20me1M84ox>	0.60%	1.90%	1.31%	3.19	0.0497

Table S9: Proteoform changes in response to HDACi (30 min) in SUM159 cells.

Proteoforms	SUM159	HDACi 30 min	Difference	Fold change	P value
<K5acK12acK16acK20me2>	0.01%	0.05%	0.04%	5.78	0.00021

<Nα-acK12acK16acK20me3K31acM84ox>	0.08%	0.28%	0.21%	3.59	0.00053
<K5acK8acK12acK16acK20me1>	0.01%	0.07%	0.06%	8.90	0.00260
<Nα-acK12acK16acK20me2>	2.29%	6.44%	4.14%	2.81	0.00335
<Nα-acK12acK16acK20me2M84ox>	0.11%	0.68%	0.57%	6.38	0.00369
<Nα-acK8acK12acK16acK20me2>	0.45%	2.67%	2.23%	5.97	0.00512
<K5acK8acK12acK16acK20me3K31ac>	0.00%	0.03%	0.03%	9.73	0.00764
<Nα-acK12acK16acK20me1M84ox>	0.01%	0.04%	0.03%	5.69	0.00899
<K5acK12acK16acK20me1>	0.02%	0.07%	0.05%	3.67	0.01691
<Nα-acK12acK16acK20me3>	0.00%	0.06%	0.06%	∞	0.02393
<Nα-acK8acK12acK16acK20me2M84ox>	0.01%	0.28%	0.28%	51.55	0.02544
<Nα-acK12acK16acK20me1>	0.51%	1.06%	0.56%	2.10	0.02762
<Nα-acK5acK8acK12acK16acK31ac>	0.00%	0.00%	0.00%	14.43	0.03174
<K5acK12acK16acK20me1K31acM84ox>	0.00%	0.00%	0.00%	5.82	0.03408
<Nα-acK5acK12acK16acK20me2M84ox>	0.00%	0.04%	0.04%	25.90	0.03825
<Nα-acK20me2>	42.26%	32.07%	-10.19%	0.76	0.04322
<Nα-acK5acK8acK12acK16acK20me2>	0.09%	1.91%	1.82%	21.53	0.04382
<Nα-acK16acK20me2>	21.84%	26.26%	4.42%	1.20	0.04469

Table S10: Proteoform changes in response to HDACi then SUV4-20 inhibition in SUM159 cells

Proteoforms	SUM159	HDACi then A196	Difference	Fold change	P value
<K5acK8acK12acK16acK20me1>	0.01%	0.08%	0.07%	9.53	0.00066
<Nα-acK12acK16acK20me1>	0.51%	1.46%	0.96%	2.89	0.00209
<K8acK20me1>	0.01%	0.01%	0.01%	2.55	0.00236
<Nα-acK8acK12acK16acK20me2>	0.45%	2.75%	2.30%	6.12	0.00465
<Nα-acK16acK20me2>	21.84%	30.26%	8.42%	1.39	0.00569
<Nα-acK5acK12acK16acK20me2>	0.10%	0.54%	0.44%	5.32	0.00612
<Nα-acK12acK16acK20me2>	2.29%	6.01%	3.71%	2.62	0.01025
<Nα-acK12acK16acK20me2K31ac>	0.51%	0.38%	-0.13%	0.75	0.01298
<Nα-acK20me2>	42.26%	30.69%	-11.57%	0.73	0.02044
<Nα-acS1phK8acK12acK16acK20me1K31ac>	0.00%	0.00%	0.00%	3.49	0.02111
<K5acK8acK12acK16acK20me2K31ac>	0.00%	0.00%	0.00%	9.02	0.02413
<Nα-acK8acK12acK16acK20me1>	0.08%	0.39%	0.31%	4.84	0.02478
<K8acK16acK20me2>	0.00%	0.01%	0.01%	4.29	0.03108
<K5acK8acK12acK16acK20me3K31ac>	0.00%	0.02%	0.02%	7.73	0.03250
<Nα-acK5acK8acK16acK20me1>	0.00%	0.02%	0.02%	19.88	0.03567
<Nα-acK5acK12acK16acK20me1>	0.01%	0.07%	0.06%	13.22	0.03576
<K5acK16acK20me2>	0.04%	0.08%	0.04%	1.93	0.04315
<K5acK16acK20me2M84ox>	0.03%	0.11%	0.07%	3.07	0.04344
<K5acK12acK16acK20me3>	0.00%	0.00%	0.00%	∞	0.04813

Table S11: Proteoform changes in response to SUV4-20 inhibition and HATi in MCF7 cells

Proteoforms	MCF7	HATi then A196	Difference	Fold change	P value
<Nα-acK12acK16acK20me2>	1.02%	3.04%	2.02%	2.98	0.00040
<Nα-acK20me2>	44.61%	28.78%	-15.84%	0.64	0.00274
<Nα-acK8acK12acK16acK20me2K31ac>	0.01%	0.10%	0.09%	10.57	0.00348
<Nα-acK8acK12acK16acK20me3K31acM84ox>	0.01%	0.02%	0.02%	4.08	0.00425
<Nα-acK16acK20me3>	0.17%	1.28%	1.11%	7.72	0.00448
<Nα-acK12acK16acK20me2K31ac>	0.22%	0.78%	0.56%	3.52	0.00885
<K5acK16acK20me3>	0.13%	0.02%	-0.11%	0.15	0.00941
<Nα-acK5acK8acK12acK16acK20me3M84ox>	0.00%	0.00%	0.00%	0.00	0.01074
<K8acK16acK20me3>	0.11%	0.00%	-0.11%	0.04	0.01090
<K5acK20me2>	0.64%	6.69%	6.05%	10.42	0.01474
<Nα-acK12acK16acK20me1K31ac>	0.03%	0.20%	0.18%	8.08	0.02169
<Nα-acS1phR3me1K5acK8acK12acK16acK20me3K31acM84ox>	0.02%	0.00%	-0.02%	0.21	0.02608
<Nα-acK8acK12acK16acK20me3K31ac>	0.02%	0.12%	0.10%	5.69	0.02835
<Nα-acK20me1>	4.25%	6.51%	2.27%	1.53	0.02928
<Nα-acK20me3>	2.31%	4.47%	2.16%	1.93	0.03242

Table S12: Proteoform changes in response to HATi in MCF7 cells

Proteoforms	MCF7	HATi	Difference	Fold change	P value
<K5acK12acK16acK20me3K31acM84ox>	0.02%	0.00%	-0.01%	0.08	0.00332
<K5acK16acK20me3>	0.13%	0.02%	-0.11%	0.17	0.00952
<Nα-acK12acK16acK20me2>	1.02%	3.38%	2.36%	3.32	0.01013
<Nα-acK5acK8acK12acK16acK20me3M84ox>	0.00%	0.00%	0.00%	0.00	0.01074
<K8acK16acK20me3>	0.11%	0.01%	-0.11%	0.04	0.01074
<Nα-acK16acK20me3>	0.17%	1.11%	0.95%	6.72	0.01473
<Nα-acK16acK20me3M84ox>	0.08%	0.00%	-0.08%	0.00	0.01762
<Nα-acK20me1>	4.25%	6.43%	2.18%	1.51	0.02007
<K5acK16acK20me2>	0.06%	0.19%	0.12%	2.96	0.02205
<Nα-acK16acK20me2>	21.75%	25.95%	4.20%	1.19	0.02298
<K5acK12acK16acK31acM84ox>	0.00%	0.00%	0.00%	0.00	0.02743
<Nα-acM84ox>	0.26%	0.00%	-0.26%	0.02	0.02872
<Nα-acS1phK8acK12acK31ac>	0.00%	0.08%	0.08%	260.29	0.03063
<Nα-acK20me3>	2.31%	3.84%	1.53%	1.66	0.03279
<Nα-acS1phR3me1K5acK8acK12acK16acK20me3K31acM84ox>	0.02%	0.00%	-0.02%	0.08	0.03281
<Nα-acK16ac>	0.06%	0.74%	0.68%	12.55	0.03539
<Nα-acK8acK12acK16acK20me3K31acM84ox>	0.01%	0.00%	-0.01%	0.06	0.03618
<K5acK16acK20me1>	0.03%	0.10%	0.07%	3.86	0.04066
<S1phK5acK8acK16acK20me3K31acM84ox>	0.00%	0.00%	0.00%	0.21	0.04380

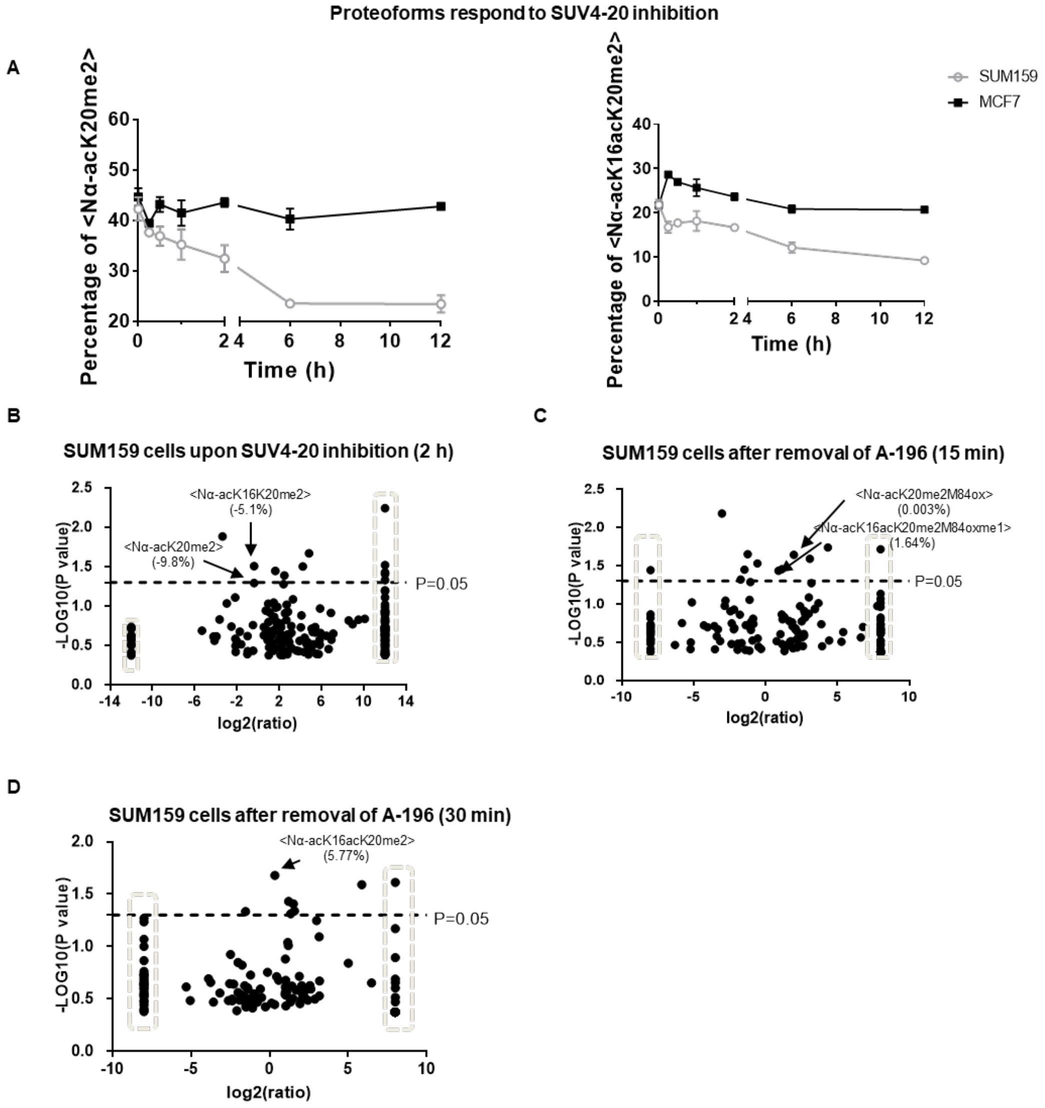

Figure S1. SUM159 and MCF7 cells respond to A-196 rapidly, and also recover immediately after removal of A-196. A) <Nα-acK20me2> and <Nα-acK16acK20me2> have different responses to SUV4-20 inhibition in SUM159 and MCF7 cells. **B)** Volcano plot of proteoform changes in response to SUV4-20 inhibition after 2 h treatment. Volcano plot of proteoforms comparison illustrates the dynamics of proteoform recovering in **(C)** 15 and **(D)** 30 min after removal of A-196. Data points in the grey square indicate infinity fold change.

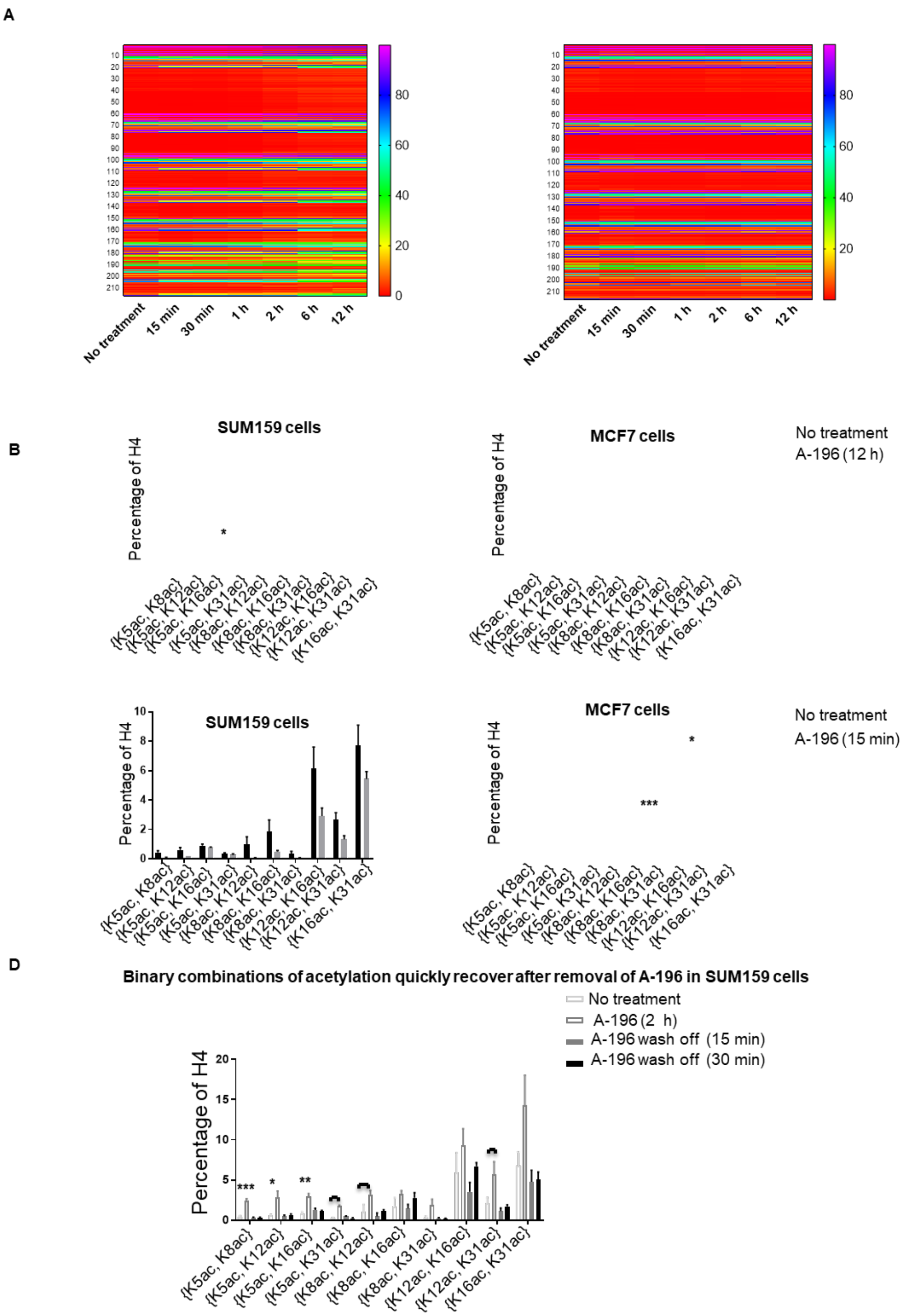

Figure S2. Binary combinations of H4 PTMs respond to SUV4-20 inhibition in both cell lines. A) Heatmap for the effect of SUV4-20 inhibition on all binary combinations of H4 PTMs. SUV4-20 inhibition causes change to specific binary combinations of acetylation in SUM159 cells and MCF7 cells after **(B)** 12 h and **(C)**15 min treatment. **D)** Increased binary combinations of H4 acetylation in SUM159 cells, due to SUV4-20 inhibition, recover in 15min. *, $p<0.05$; **, $p<0.01$.

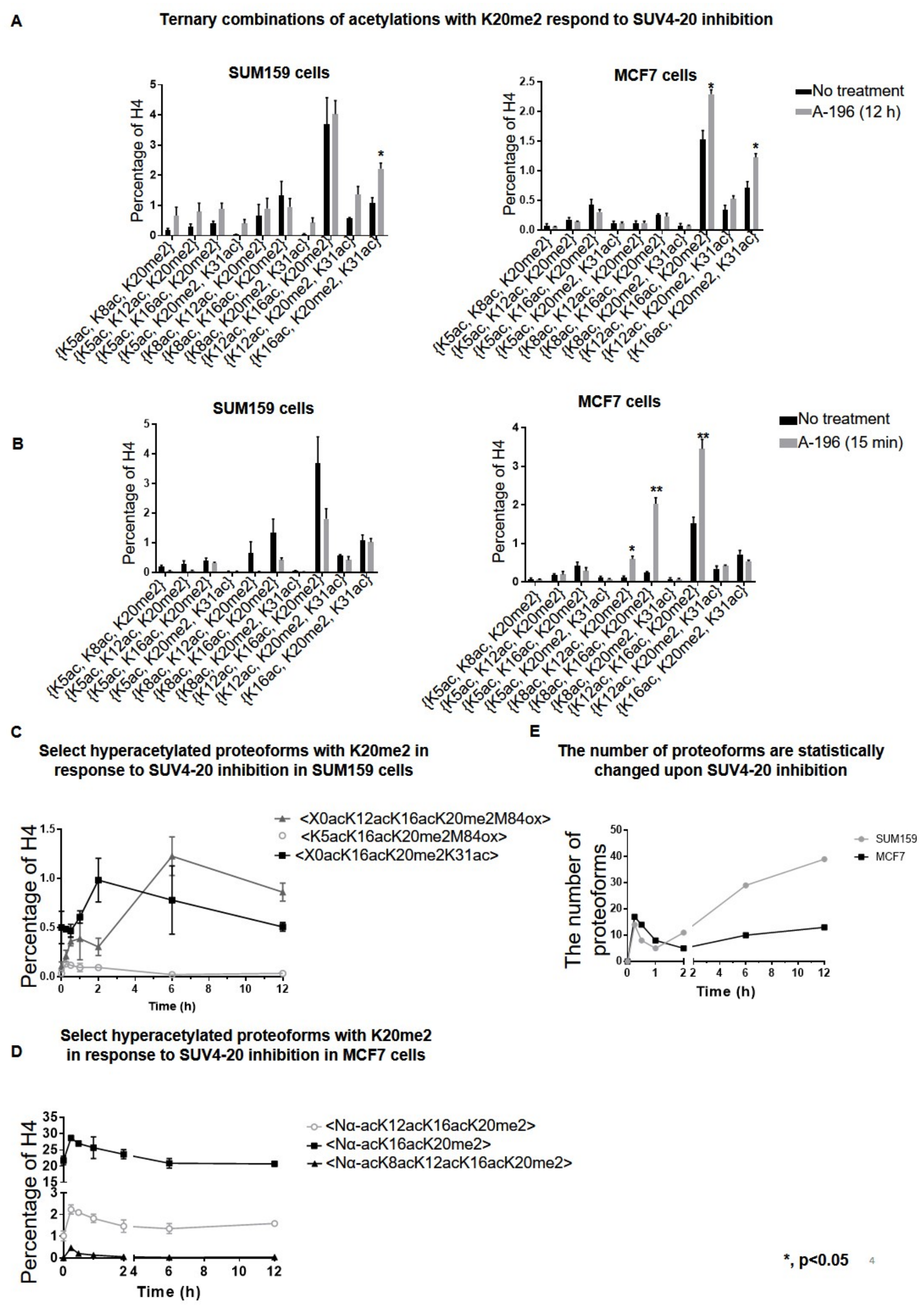

Figure S3. Positive correlation between K20me2 and H4 acetylation. The effect of SUV4-20 inhibition on ternary combinations containing K20me2 and two acetylations in SUM159 and MCF7 cells after **(A)** 12 h and **(B)** 15 min treatment. The dynamics of hyperacetylated proteoforms with K20me2 upon SUV4-20 inhibition in (**C**) SUM159 and (**D**) MCF7 cells. **E)** The number of proteoforms statistically affected by SUV4-20 inhibition at each time point in SUM159 and MCF7 cells ($p<0.05$). *, $p<0.05$

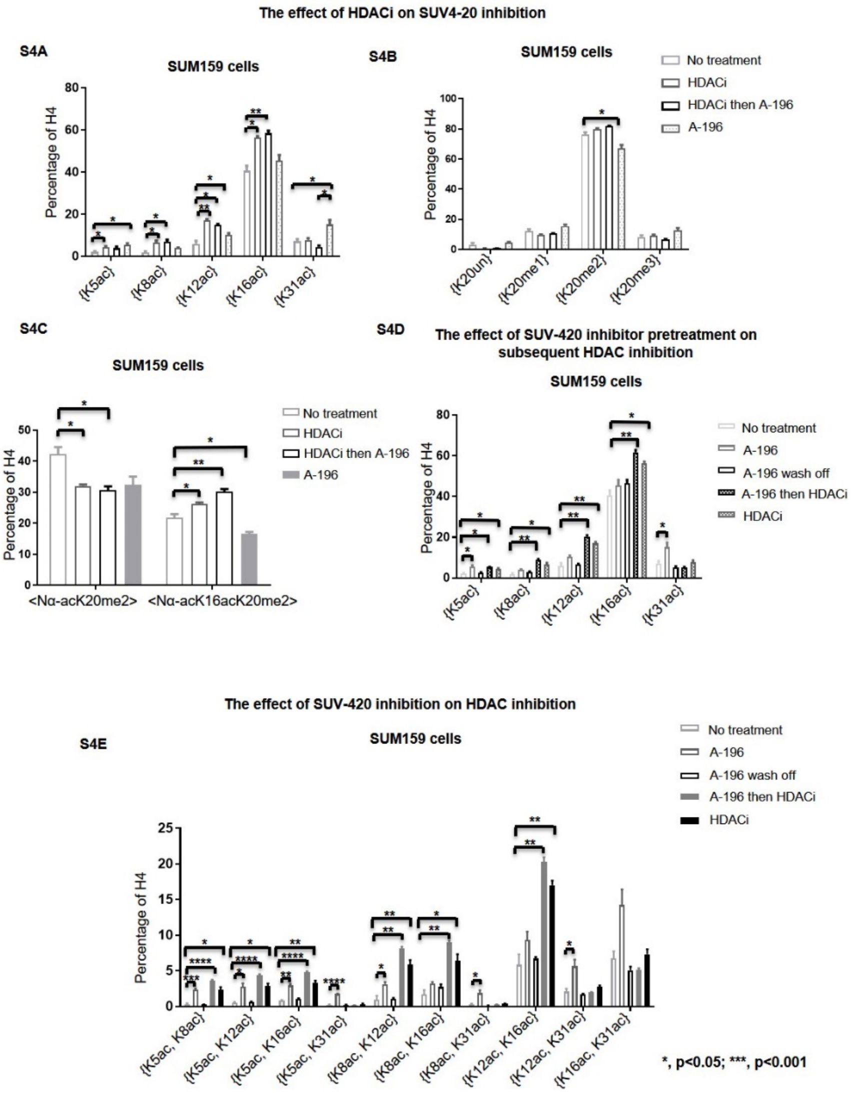

5

Figure S4. HDAC inhibition substantially diminishes the effect of SUV4-20 inhibition, and SUV4-20 inhibition stimulates HDACi in SUM159 cells. The effect of SUV4-20 inhibition, HDAC inhibition or HDACi then SUV4-20 inhibition on **(A)** discrete H4 acetylations, **(B)** H4K20 methylation states, **(C)** top two abundant proteoforms. The effects of SUV4-20 inhibition then HDACi on **(D)** discrete and **(E)** binary combination of H4 acetylations. *, p<0.05; **, p<0.01.

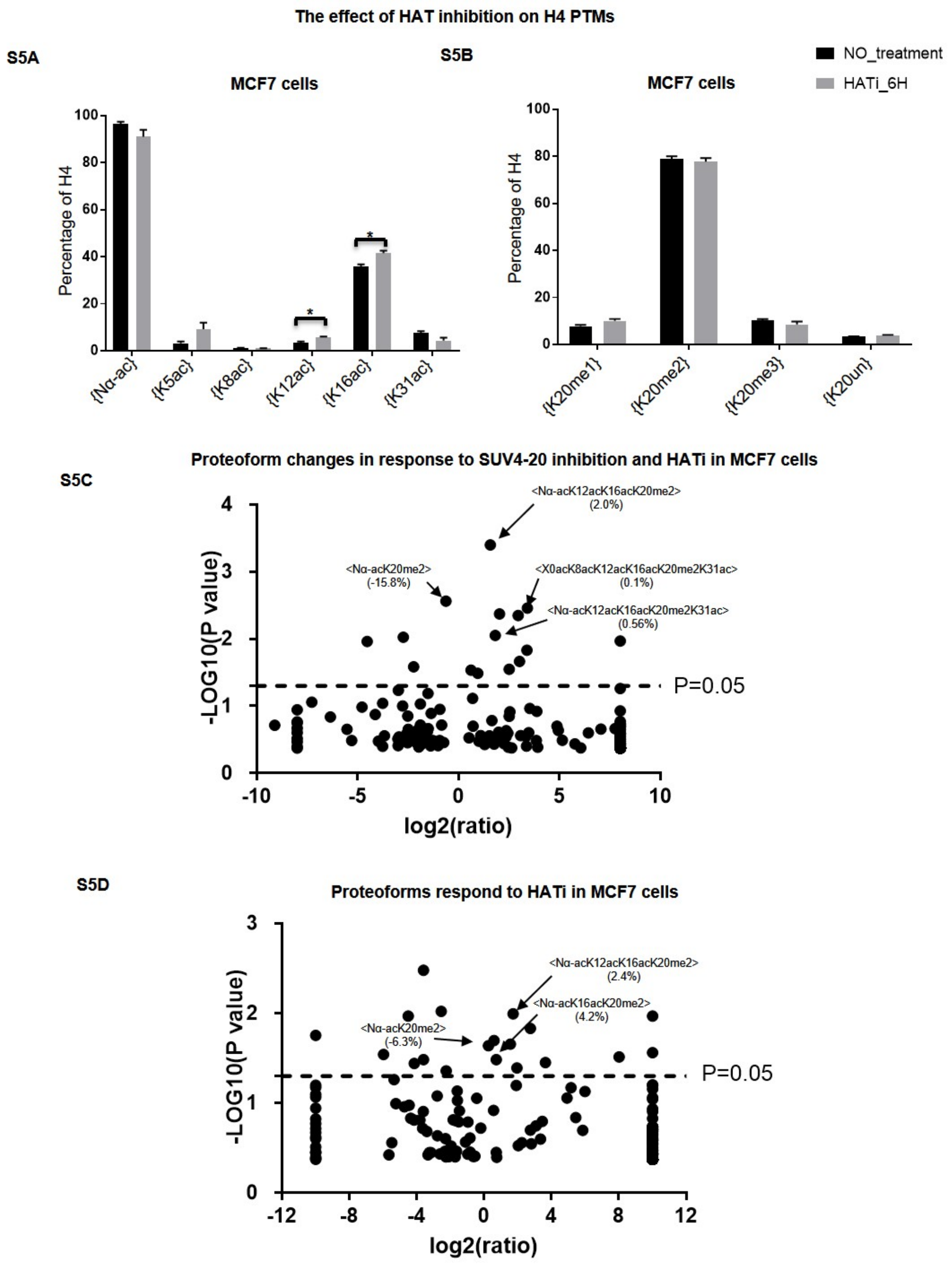

Figure S5. SUV4-20 stimulates HAT activity. The effects of HATi on **(A)** discrete H4 acetylations and **(B)** discrete H4{K20} methylation states**. C)** Volcano plot of proteoforms change upon HATi and SUV420 inhibitor application **D)** Volcano plot of proteoforms change upon HATi application. Data points in the grey square indicate infinity fold change. *, p<0.05; **, p<0.01.
